# Reactive ZIF‑L Crystal Surface for Organophosphorous Degradation and Acetylcholinesterase Reactivation

**DOI:** 10.1021/jacs.5c00382

**Published:** 2025-03-10

**Authors:** Emilio Borrego-Marin, Pablo Garrido-Barros, Gregory W. Peterson, Rebecca Vismara, Francisco J. Carmona, Elisa Barea, Jorge A. R. Navarro

**Affiliations:** † Departamento de Química Inorgánica, 16741 Universidad de Granada, Av. Fuentenueva S/N, 18071, Granada, Spain; ‡ U.S. Army Combat Capabilities Development Command Chemical Biological Center, 8198 Blackhawk Road, Aberdeen Proving Ground, Maryland 21010, United States

## Abstract

The importance of crystal surface reactivity of reticular materials is exemplified by exfoliation of nonporous layered zeolitic imidazolate framework Zn­(mIm)_2_·0.5mImH (ZIF-L, mImH = 2-methylimidazole). Sonication of ZIF-L ethanolic suspensions leads to exfoliation of microcrystals along the 2 0 0 planes, giving rise to 1.5 μm wide × 25 nm thick flakes, which we term ZIF-L_exf. ZIF-L_exf exhibits a high reactivity toward hydrolytic degradation of extremely toxic G-type nerve agents, Soman (GD), and simulant diisopropylfluorophosphate (DIFP). The reactivity of the crystal surface of ZIF-L_exf toward P–F bond breakdown gives rise to framework structural degradation, releasing nucleophilic mImH molecules that reactivate organophosphate-inhibited acetylcholinesterase within 10 min. This detoxification process can be taken as a proof of concept for reversing organophosphorous poisoning. More generally, this approach underscores the importance of the crystal surface nature and composition to control the reactivity of reticular materials.

## Graphical abstract

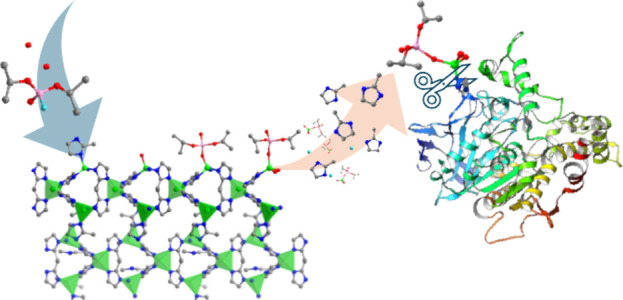

Reticular chemistry has boosted the design of crystalline frameworks based on the simple principles of directional reversible bonds, leading to unlimited compositions, structures, and porosities, suited for a paramount variety of properties and applications.^1–6^ A general assumption of reticulated porous materials is that the primary interaction site with a substrate is the pore surface/void space.^7–10^ In this regard, while the bulk and pore nanospace properties of these materials have been extensively studied, the chemistry of the external crystal surface of reticular materials remains underexplored. It should be highlighted that the crystal surface determines myriads of fundamental processes: for instance, in living systems the interaction of a crystal surface with biomolecules and cells determines the formation of bone tissue,^11,12^ biomineral shells,^13^ and gout pathologies.^14^ Moreover, toxicity of the contact insecticide DDT^15,16^ and activity of pharmaceutical drugs (polymorphism) are determined by the crystal phase/crystal face.^17^ Similarly, crystal surface reactivity determines the catalytic activity of nonporous catalysts (i.e., platinum group metals).^18,19^

In this communication, we have selected nonporous layered zinc imidazolate framework Zn­(mIm)_2_·0.5mImH (ZIF-L, mIm = 2-methylimidazolate)^20–22^ as an example of a reticulated material in which we propose its reactivity is mainly dictated by the crystal surface (Scheme 1). To demonstrate our hypothesis, ZIF-L microcrystals have been exfoliated, by sonication, along the 2 0 0 crystallographic planes to increase the crystal reactivity. The resulting flakes exhibit an enhanced hydrolytic degradation reactivity toward real G-type nerve agent 3,3-dimethyl-2-butanylmethyl­phosphonofluoridate (Soman, GD) and simulant diisopropyl­fluorophosphate (DIFP).^23,24^ The reactivity of the crystal surface toward these toxic compounds gives rise to framework structural degradation with concomitant release of nucleophilic 2-methylimidazole linkers, which in turn reactivate phosphorylated acetylcholinesterase (OP@AChE) adduct, thereby reversing organophosphorus poisoning (Scheme 1b).

**Scheme 1:**
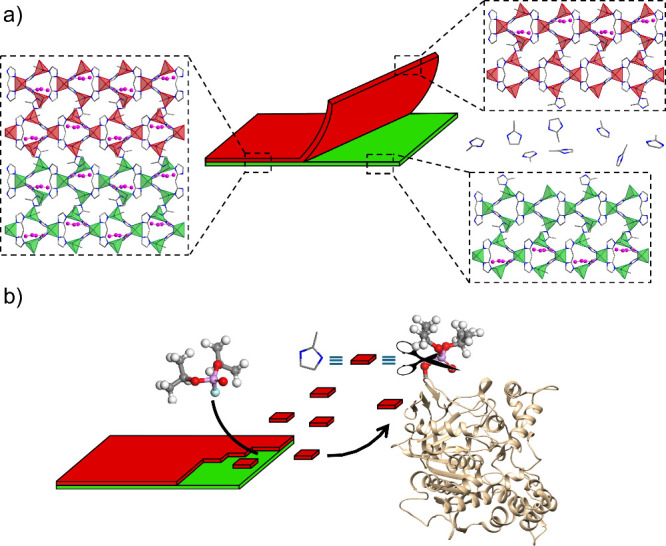
Exfoliation and Crystal Surface Reactivity of ZIF-L for Nerve Agent Detoxification: (a) Schematic Representation of Zn­(mIm)_2_·0.5mImH (ZIF-L) Framework Exfoliation along 2 0 0 Crystallographic Planes by Sonication of Ethanolic ZIF-L Suspensions; (b) Nerve Agent Induced Crystal Surface Degradation of ZIF-L Leading to Phosphorylated Acetylcholinesterase (OP@AChE) Reactivation by Nucleophilic Attack of Released Imidazole Moieties^a^

a Color code: C (gray), F (light blue), H (white), N (blue), O (red), P (magenta). Zn^2+^ ions have been represented as green and red tetrahedra to differentiate two consecutive Zn­(mIm)_2_ layers in the ZIF-L crystal structure.

Microcrystals of ZIF-L, 3.0 μm wide × 250 nm thick (Figures 1, S1), have been prepared using literature methods.^25^ Sonication of the ZIF-L ethanolic suspension (2.5 mg/mL) gives rise to the formation of a stable colloidal suspension of exfoliated ZIF-L crystals that we term ZIF-L_exf (Figures 1, S2–S5). Drop casting of the ZIF-L_exf suspension, on a silicon wafer, gives rise to 2*n* 0 0 high-intensity peaks in the powder X-ray diffraction (PXRD) pattern, indicative of a high preferential orientation along the crystallographic *a* axis (Figure 1a). N_2_ adsorption experiments at 77 K for ZIF-L and isolated ZIF-L_exf flakes give rise to a 3-fold increase in adsorption capacity with the BET surface passing from 11 m^2^ g^–1^ to 37 m^2^ g^–1^. Considering the nonporous nature of ZIF-L, the higher surface area is indicative of an improved external particle surface accessibility and/or higher concentration of surface defects upon exfoliation (Figure 1b). Scanning electron microscopy (SEM) images of ZIF-L (3 μm wide × 250 nm thick) after 5 min of sonication lead to the formation of thin flakes of ZIF-L_exf (1.5 μm wide × 25 nm thick) (Figures 1c,d, S4). These results agree with crystal exfoliation along the 2 0 0 crystallographic planes containing clathrated mImH molecules corresponding to approximately 20 molecular layer stacks (∼20 times half of the crystallographic *a* axis).

**Figure 1:**
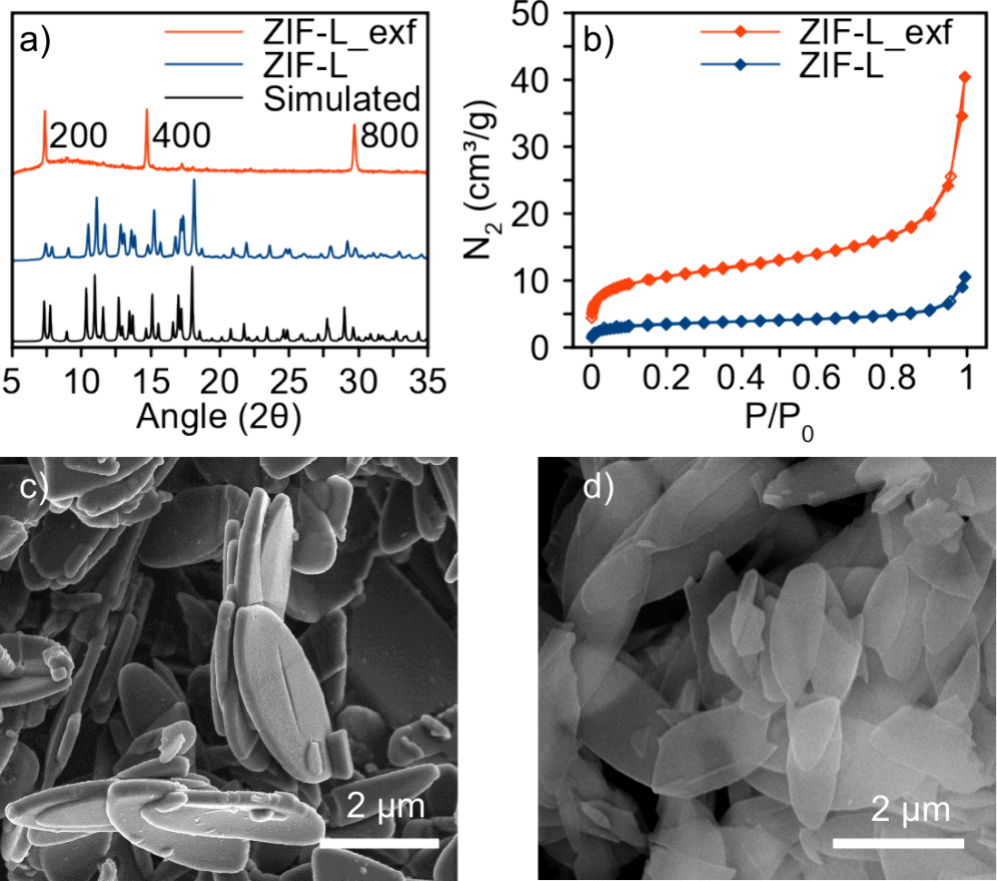
Characterization of ZIF-L and ZIF-L_exf: (a) Powder X-ray diffraction of pristine ZIF-L (experimental, blue; calculated black) and exfoliated ZIF-L_exf (red). (b) Nitrogen adsorption isotherms at 77 K for pristine ZIF-L (blue) and ZIF-L_exf (red). Scanning electron microscopy images of (c) ZIF-L and (d) ZIF-L_exf.

Following structural characterization of the ZIF-L_exf system, we have explored the impact of ZIF-L exfoliation on crystal surface reactivity toward G-type real nerve agent Soman (GD) and simulant diisopropyl­fluorophosphate (DIFP) hydrolytic degradation. We term this process *nerve agent decontamination*. In the following step, we have studied the release of imidazole nucleophile, from ZIF-L_exf crystal face structural degradation, which reactivates organophosphorus-inhibited acetylcholinesterase. We term this process *nerve agent detoxification*.

The reactivity of ZIF-L and ZIF-L_exf (0.084 mmol) toward the model nerve agent DIFP (0.029 M, 0.5 mL) was essayed under simulated biological conditions (Tris-HCl, 0.1 M, pH = 7.4) (Figure 2a). The results indicate that ZIF-L and ZIF-L_exf can hydrolytically break down the P–F bond of DIFP, yielding nontoxic diisopropylphosphate (DIP) (Figure 2a). Half-life times *t*_1/2_ for DIFP degradation of 27 min (*k* = 2.57 × 10^–2^ min^–1^) for ZIF-L diminishes to 8 min (*k* = 8.66 × 10^–2^ min^–1^) for ZIF-L_exf, with 100% degradation being achieved after 25 min (Figures 2b, S6–S8).

**Figure 2:**
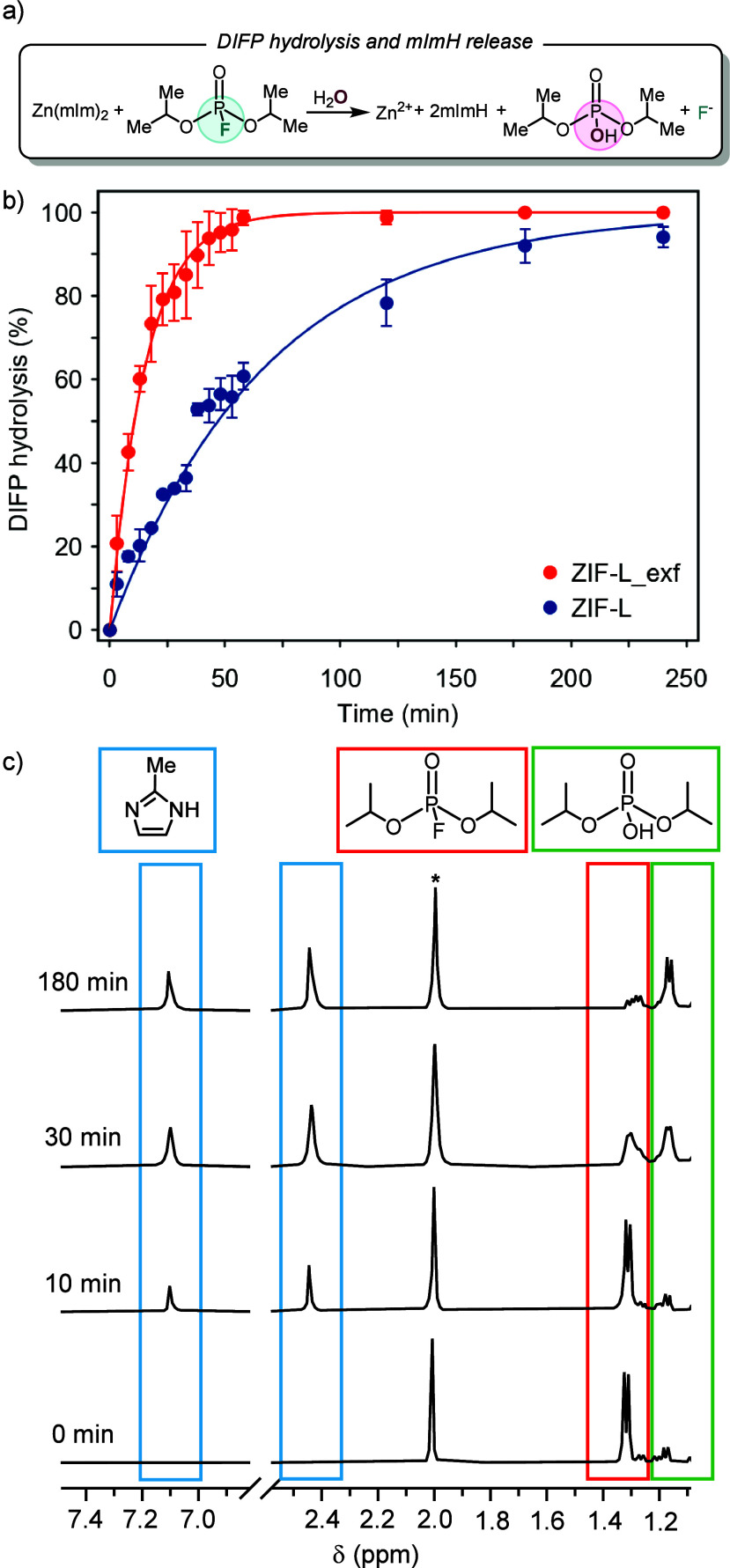
ZIF-L reactivity toward model G-type nerve agent DIFP: (a) Schematic of DIFP-induced degradation of the ZIF-L_exf crystal surface leading to hydrolytic breakdown of DIFP into nontoxic diisopropylphosphate (DIP) and concomitant release of framework structural components. (b) Profiles of DIFP (0.029 M, 0.5 mL) hydrolytic degradation, under simulated biological conditions (Tris-HCl, 0.1 M, pH = 7.4), showing enhanced crystal surface reactivity of ZIF-L upon exfoliation into ZIF-L_exf (0.084 mmol). (c) ^1^H NMR follow-up of the coupled DIFP breakdown and ZIF-L_exf structural degradation with concomitant mIm release. Experimental conditions: 0.5 mL of Tris-DCl (0.1 M, pD = 7.0), 0.015 mmol of DIFP, 0.015 mmol of dimethylacetamide (internal standard denoted with an asterisk in the spectra), and 0.084 mmol of ZIF-L_exf.

DIFP reaction with the ZIF-L and ZIF-L_exf crystal surface also triggers framework structural degradation with 2-methylimidazole linker release, as proven by ^1^H NMR (Figures 2c, S9, S10). AFM images also confirm DIFP-induced particle surface etching leading to a decrease in flake thickness (Figure S11). Furthermore, *in situ* Raman spectra of DIFP-induced ZIF-L crystal surface degradation show significant intensity lowering of peaks at 1180 and 1455 cm^–1^, tentatively associated with the loss of terminal mImH residues, together with the appearance of signals at 920 and 3450 cm^–1^ associated with the incorporation of organohosphate and hydroxide residues, respectively (Figure S12). Moreover, hot filtration tests also confirm the heterogenicity of crystal reactivity (Figures S13, S14). This is further substantiated with control reactions using Zn­(NO_3_)_2_ and mImH in Tris-HCl, 0.1 M (pH = 7.4) that do not yield any significant DIFP hydrolysis (Figure S15). Recyclability tests for ZIF-L and ZIF-L_exf indicate that these materials are still active for DIFP degradation on successive cycles (Figures S16, S17). It is noteworthy that ZIF-L_exf suffers from significant successive weight losses of 60% and 50% after the first and second cycle, respectively. Characterization of the materials after 4 cycles of DIFP degradation showed that the ZIF-L crystalline phase is maintained, although the appearance of FTIR bands in the infrared spectrum associated with the (organo)­phosphate group are noticed and in agreement with the above-described *in situ* Raman studies (Figures S18–S21). Considering the nonporous nature of the ZIF-L framework, the improved reactivity for ZIF-L_exf must be related to the increased exposed external crystal surface.

A second decontamination experiment was carried out impregnating the surface of the materials (0.084 mmol) with real nerve agent Soman (GD) or simulant DIFP (0.015 mmol) under 50% relative humidity at room temperature to simulate the behavior of a protective filter. The evolution of the hydrolysis reaction was quantified by solid–liquid extraction with acetonitrile (1 mL) at different timeframes (Figures S22–26, Table S1). The results show that ZIF-L exfoliation increases the kinetics constant for DIFP hydrolysis from 0.020 h^–1^ (*t*_1/2_ = 34.7 h) for ZIF-L to 0.057 h^–1^ (*t*_1/2_ = 12.2 h) for ZIF-L_exf (Figures S22–24). Following a similar procedure but using GD instead of simulant, ZIF-L_exf also outperforms ZIF-L for real nerve agent degradation with respective *k* values of 0.206 h^–1^ (*t*_1/2_ = 3.4 h) and 0.161 h^–1^ (*t*_1/2_ = 4.3 h) and quantitative degradation being reached after 24 h (Figures S25, S26, Table S1). The lower kinetics for DIFP degradation in comparison to GD is consistent with the higher reactivity of real nerve agents in comparison to simulants.

DFT calculations have been carried out to interrogate the DIFP-induced ZIF-L_exf crystal surface structural breakdown. [Zn­(mImH)_4_]^2+^ has been selected as a simplified model of the ZIF-L­(_exf) reactive surface (Figure 3). The results are indicative of a thermodynamically favorable 3-step pathway consisting of (i) a ligand exchange process of a terminal mImH ligand by a hydroxide ion (Δ*G* = −14.9 kcalmol^–1^); (ii) P–F bond hydrolysis on an activated Zn-hydroxide center, leading to a coordinated DIP fragment (Δ*G* = −18.2 kcalmol^–1^). A transition state for nucleophilic attack of the Zn-hydroxide residue to a phosphorus atom (Δ*G* ^⧧^ = +13.1 kcalmol^–1^) has been modeled (Figure S27, Movie S1). (iii) mImH ligand exchange by a hydroxide ion to hydrolytically break down the ZIF-L_exf crystal surface (Δ*G* = −15.4 kcalmol^–1^). Alternatively, DIP ligand exchange by a hydroxide ion to regenerate a Zn-hydroxide active center is also thermodynamically favored, although to a lower extent (Δ*G* = −10.2 kcal mol^–1^). It is noteworthy that the latter process becomes more favorable with the progress of the structural degradation (see Figure S28).

**Figure 3:**
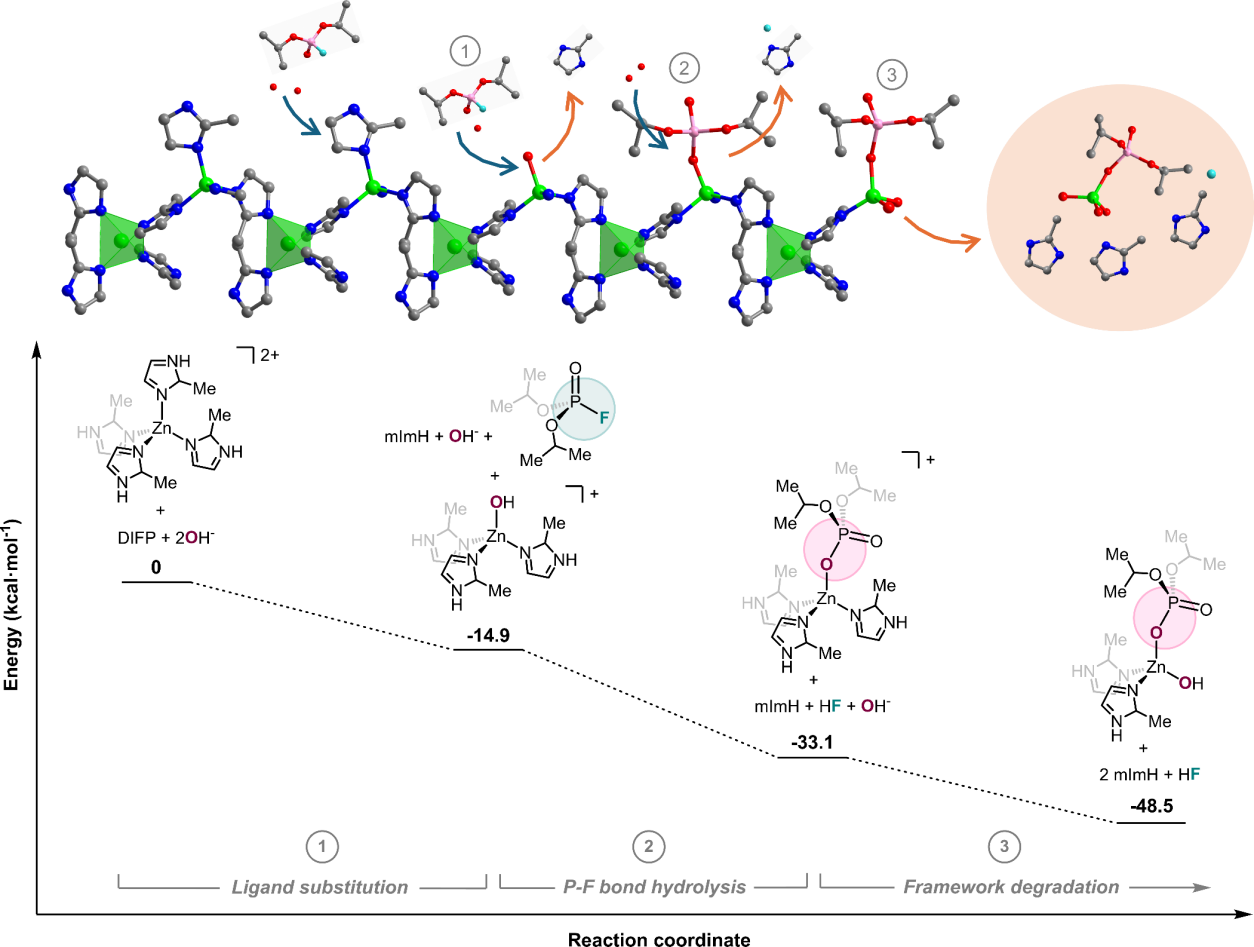
DFT calculation of the energy profile for the DIFP-induced ZIF-L_exf crystal surface degradation: (i) Hydrolytic breakdown of the terminal Zn–mIm bond leading to Zn–OH reactive centers; (ii) P–F bond hydrolysis leading to attached DIP residue; (iii) hydrolytic breakdown of Zn–mImH bonds leading to the structural framework degradation.

We have previously demonstrated the dual benefit of nerve agent simulant (DIFP) hydrolysis and framework structural degradation inducing nucleophilic 2-methylimidazole reactivation of phosphorylated AChE by microporous sod-ZIF-8 nanoparticles.^26^ Considering this background, we have evaluated the enhanced crystal surface reactivity of nonporous ZIF-L, upon exfoliation, for dual nerve agent hydrolysis and reactivation of phosphorylated AChE (Figure 4a).

**Figure 4:**
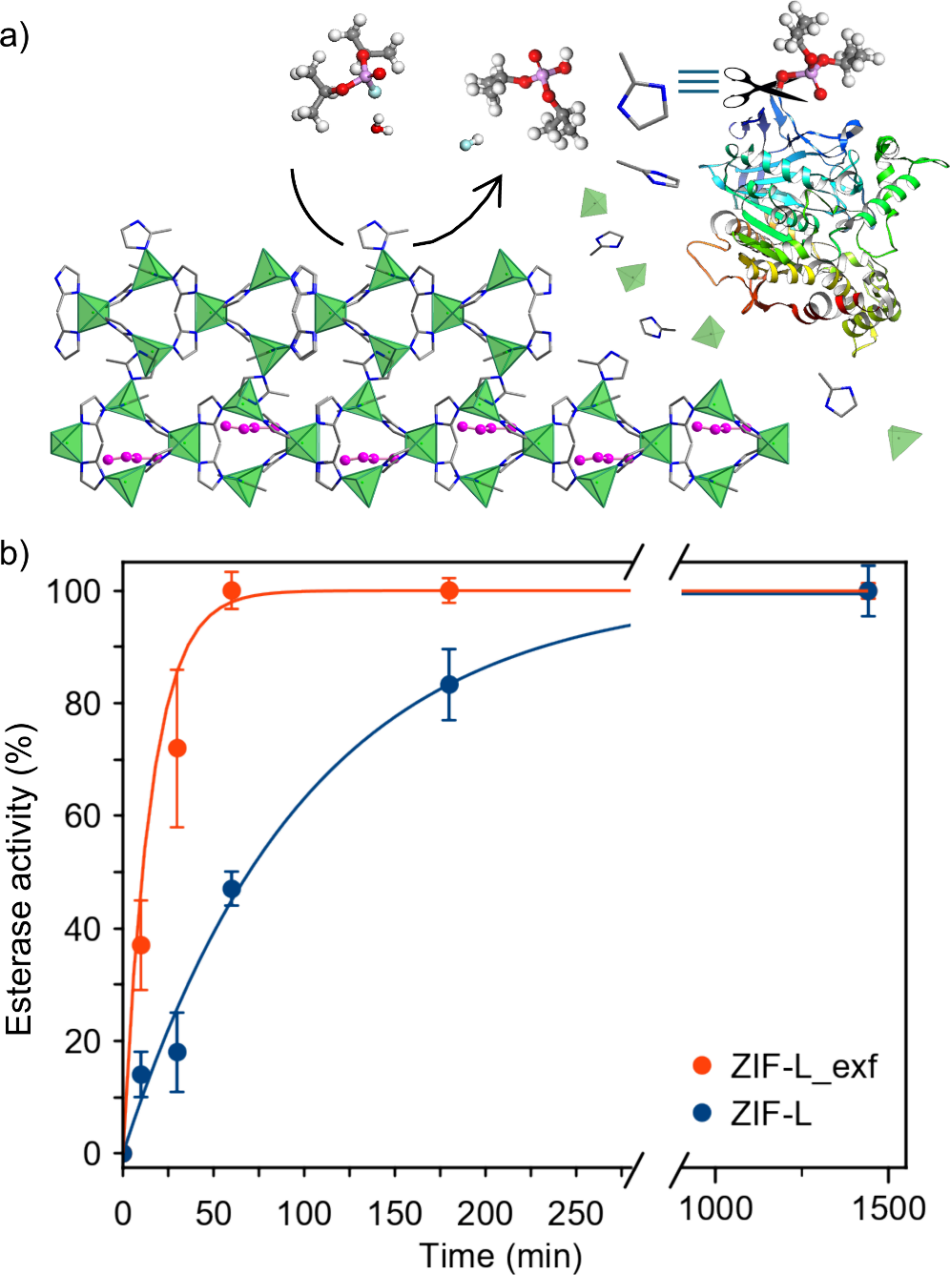
Nerve agent detoxification: (a) Schematic representation of the DIFP-induced crystal surface structural degradation with concomitant release of mIm nucleophile able to reactivate phosphorylated AChE. (b) Detoxification capacity of ZIF-L­(_exf) materials toward DIFP inhibitory effect on AChE esterase activity (Tris-HCl, 0.1 M, pH = 7.4).

We first evaluated the AChE inhibition with model nerve agent DIFP and its subsequent reactivation with free mImH nucleophile under simulated biological conditions (Tris-HCl, 0.1 M, pH = 7.4). DIFP has an important inhibition effect on AChE esterase activity above concentrations as low as 1 × 10^–7^ M and quantitative inhibition above 1 × 10^–4^ M (see Figure S29). Subsequently, we evaluated the ability of mImH to reactivate a 50% inhibited AChE ([DIFP] = 5 × 10^–6^ M). The results show that mImH concentrations of this nucleophile above 1 × 10^–4^ M show an incipient reactivation ability with quantitative reactivation being achieved at 1 × 10^–1^ M (Figure S30). The latter value falls in the range of the concentration of mImH released upon incubation of ZIF-L­(_exf) materials with DIFP (Table S2).

In a second step, we investigated the dual *nerve agent decontamination and detoxification* ability of ZIF-L and ZIF-L_exf materials. With this aim, ZIF-L and ZIF-L_exf particles (0.084 mmol) were dispersed in a DIFP solution (0.029 M, 0.5 mL) under simulated biological conditions (Tris-HCl, 0.1 M, pH = 7.4) and subsequently incubated at 37 °C for 10, 30, 60, 180, and 1440 min (see Supporting Information). Afterward, the supernatants were collected from ZIF suspensions, containing released mImH from framework structural degradation (Table S2), and evaluated for the mitigation of the inhibitory effect of DIFP on AChE activity (Figure 4). The detoxification profiles exerted by ZIF-L and ZIF-L_exf (Figure 4b) follow a trend similar to that of DIFP hydrolytic breakdown (Figure 2b). Indeed, ZIF-L_exf flakes can recover 37% of AChE esterase activity within 10 min and quantitatively within 1 h (Figure 4b), because of a synergistic combination of DIFP hydrolysis and released mImH, from crystal surface structural degradation. By contrast, ZIF-L exhibits a poorer detoxification profile, with quantitative enzymatic activity recovery being reached only after 24 h of incubation (Figure 4b). Moreover, a control DIFP solution (0.029 M, 0.5 mL), in the absence of ZIF-L­(_exf) crystals, leads to quantitative inhibition of AChE esterase activity. These results highlight the benefits of DIFP hydrolysis and mImH release taking place at the ZIF-L crystal surface for simultaneous *nerve agent decontamination* and *detoxification*. This approach offers a more straightforward method than our previous reports relying on the hydrolytic activity of highly acidic centers (i.e., Zr) and delivery of pore-encapsulated oxime drugs.^27,28^ The latter is affected by the limited loading of active drugs in the pores and the lack of control over their release.

As a summary, we have shown the importance of increasing ZIF-L 2 0 0 crystal face reactivity by materials exfoliation. As a more general take-home message, we underscore the importance of crystal surface nature and composition to control the reactivity of reticular materials.

## Supplementary Material





## Data Availability

Optimized structures from DFT calculations with the corresponding Cartesian coordinates are available at ioChem database at: http://dx.doi.org/10.19061/iochem-bd-6-446.

## Notes

Synthetic protocols, full characterization of ZIF-L materials, DFT calculations, details of DIFP detoxification, AChE inhibition and reactivation assays; additional references (PDF)
DFT calculation of transition state for nucleophilic attact of Zn-OH residue of ZIF-L surface to P–F bond of nerve gas surrogate (MOV)
