# Supplementary material for: Reactive ZIF‑L Crystal Surface for Organophosphorous Degradation and Acetylcholinesterase Reactivation
Source: J Am Chem Soc. 2025 Mar 10;147(13):10834–9. doi: 10.1021/jacs.5c00382 (PMC12150325; doi:10.1021/jacs.5c00382)
Supplement: Supplementary file 1 [file ja5c00382_si_001.pdf]

Reactive ZIF-L Crystal Surface for Organophosphorous Degradation and Acetylcholinesterase Reactivation

Emilio Borrego-Marin [a], Pablo Garrido-Barros[a], Gregory W. Peterson[b], Rebecca Vismara[a], Francisco J. Carmona[a], Elisa Barea[a], and Jorge A. R. Navarro[a]

[a] Departamento de Química Inorgánica, Universidad de Granada, Av. Fuentenueva S/N, 18071, Granada, Spain.

E-mail: jarn@ugr.es

[b] U.S. Army Combat Capabilities Development Command Chemical Biological Center 8198 Blackhawk Road, Aberdeen Proving Ground, MD 21010, USA.

Table of Contents

S1. General Methods and Materials 3
S2. Synthetic Procedures. 4
S2.1. Synthesis of ZIF-L 4
S2.2. Preparation of ZIF-L exfoliated 4
S3. G-type nerve agent simulant degradation in Tris-HCl buffered solution (pH = 7.4) .. 4
S3.1. ZIF-L(_exf) 4
S3.2. Control reactions with $Zn(NO_3)_2 \cdot 6H_2O$ and mImH 4
S3.3. Hot filtration test. 4
S3.4. Recyclability assays. 4
S4. Enzymatic assays 5
S4.1. AChE inhibition 5
S4.2. AChE reactivation by 2-methylimidazole 5
S4.3. Detoxification experiments 5
S5. G-type nerve agent simulant degradation with Dose-Extraction method 6
S6. Real nerve agent degradation with Dose-Extraction method 6
S7. DFT calculations 6
S8. Figures 7
S9. Tables 34
S10. References 35

S1. General Methods and Materials

Materials and reagents. All chemical reagents and solvents were commercially obtained from Sigma-Aldrich and were used without further purification.

Powder X-Ray diffraction (PXRD) analysis. PXRD data were collected on a Bruker D2 PHASER diffractometer equipped with a Cu anode (Kα radiation, λ= 1.5418 Å), collecting with a 1mm slit in the 5-35° 2θ range with steps of 0.02° and a time between each step of 1 s.

Nitrogen adsorption isotherms. Nitrogen adsorption isotherms were measured at 77 K on a Micromeritics 3Flex volumetric instrument. Prior to measurement, samples were heated at 373 K for 12 h and outgassed to 10^{-1} Pa in a Micromeritics SmartVacPrep Module.

Scanning Electron Microscopy (SEM) analysis. SEM images were obtained on a JEOL JSM-6490LV Microscope system from the Central Research Support Services of the University of Malaga.

Z potential analysis. Z potential data was obtained on a Litesizer DLS 500 Anton-Paar employing Omega Cuvettes made from polycarbonate and filled with 350 µL of sample.

Raman spectroscopy. Raman analysis were carried out in a high-performance microRaman system HORIBA XploRA™ PLUS.

Atomic Force Microcopy (AFM). AFM images were collected on a NX20 (Park Systems) located in the scientific instrumentation center of the University of Granada. Prior to being mounted in the AFM, the basal plane of the mica sample was freshly cleaved using a sticky tape in a dust free, horizontal laminar flow hood. Samples were dropwise deposited on this surface using 30 µL of suspension. In order to facilitate particle dispersion during deposition, the mica sample holder was rotated during deposition in a spin-coater.

Sonication process. All exfoliation procedures were carried out using a BANDELIN SONOPULS mini20 equipped with an ultrasonic transducer and a microtip that is directly immersed in the suspension material.

Diisopropylfluorophosphate (DIFP) degradation experiments. Simulant nerve agent degradation experiments were followed in an Agilent 8860 Gas Chromatograph. This chromatograph has a 16 port autosampler, a HP-5 column (50 m length, 0.320 mm diameter and 1.05 µm thickness) and a FID detector.

Soman (GD) degradation experiments. Soman (GD) is a lethal chemical that should only be handled by trained and authorized personal. GD degradation process was performed in the U.S. Army Combat Capabilities Development Command Chemical Biological Center and determined by an Agilent 6890 gas chromatograph equipped with a 5973 mass spectrometer.

Acetylcholinesterase (AChE) activity determination. AChE activity was evaluated by using a colorimetric method which employs indoxyl acetate as a substrate that is converted into indigo blue. The concentration of the enzymatic product was calculated by UV−vis spectroscopy at λ = 620 nm using a Nanoquant model Inifinite M200 Pro with 24-well plate.

^{1}H and ^{31}P Nuclear Magnetic Resonance Spectroscopy (NMR). NMR data was recorded on a 400 MHz BRUKER Nanobay Avance III HD High-Definition spectrometer.

S2. Synthetic Procedures.

S2.1. Synthesis of ZIF-L

0.540 g of zinc nitrate hexahydrate, $Zn(NO_3)_2 \cdot 6H_2O$, (1.8 mmol) were dissolved in 40 mL of milli-Q water and 1.3 g of 2-methylimidazole (15.8 mmol) were dissolved in a separate solution of 40 mL of milli-Q water. Both solutions were mixed and stirred during 24 hours at room temperature. The resulting white solid was washed with water (3 x 20 mL) and recovered by centrifugation (4000 rpm, 10 min).[29]

S2.2. Preparation of ZIF-L exfoliated

ZIF-L was exfoliated by suspending 10 mg of freshly prepared ZIF-L in 4 mL of ethanol and sonicated during 5 minutes using an ion probe sonicator maintaining the temperature at 0 ºC using an ice bath. This material is denoted as ZIF-L_exf.

S3. G-type nerve agent simulant degradation in Tris-HCl buffered solution (pH = 7.4)

S3.1. ZIF-L(_exf)

The hydrolytic degradation of nerve agent simulant diisopropylfluorophosphate by ZIF-L(_exf) materials was evaluated using gas chromatography analysis. In a standard experiment, ZIF-L(_exf) (0.084 mmol) was combined with DIFP (2.5 μL, 0.015 mmol) and dimethylformamide (DMF, 1.08 μL, 0.015 mmol, used as an internal reference) in 0.5 mL of Tris−HCl buffer solution (0.1 M, pH = 7.4). The mixture was stirred at room temperature, and the change in DIFP concentration was tracked via gas chromatography and ^{1}H-NMR analysis.

S3.2. Control reactions with $Zn(NO_3)_2 \cdot 6H_2O$ and mImH

Additional experiments were carried out with mImH and $Zn(NO_3)_2 \cdot 6H_2O$ precursors as control. In this case, we selected mImH concentration corresponding to that released by ZIF-L at 24 hours. In a standard experiment, mIm (0.01 mmol) or $Zn(NO_3)_2 \cdot 6H_2O$ (0.01 mmol) were combined with DIFP (2.5 μL, 0.015 mmol) and dimethylformamide (DMF, 1.08 μL, 0.015 mmol, used as an internal reference) in 0.5 mL of Tris−HCl buffer solution (0.1 M, pH = 7.4). The mixture was stirred at room temperature, and the change in DIFP concentration was followed via gas chromatography analysis.

S3.3. Hot filtration test.

In order to investigate the heterogeneous nature of DIFP degradation by ZIF-L(_exf) materials. In a typical experiment, ZIF-L(_exf) (0.084 mmol) was combined with DIFP (2.5 μL, 0.015 mmol) and dimethylformamide (DMF, 1.08 μL, 0.015 mmol, used as an internal reference) in 0.5 mL of Tris−HCl buffer solution (0.1 M, pH = 7.4). The mixture was stirred at room temperature, and the change in DIFP concentration was tracked via gas chromatography analysis. When DIFP degradation reaches approximately 50 %, we filter the reaction to separate the material, and the reaction filtrate is monitored over time.

S3.4. Recyclability assays.

Recyclability of the materials was studied in terms of their ability to degrade DIFP in suspension by adding successive pulses of the nerve agent. In a typical experiment, ZIF-L(_exf) (0.084 mmol) was combined with DIFP (2.5 μL, 0.015 mmol) and dimethylformamide (DMF, 1.08 μL, 0.015 mmol, used as an internal reference) in 0.5 mL of Tris−HCl buffer solution (0.1 M, pH = 7.4). The mixture was stirred at room

temperature, and DIFP degradation was quantified every 4 hours via gas chromatography analysis. Between each cycle the material is washed in milli-Q water.

S4. Enzymatic assays

The enzymatic activity was assessed by the indoxyl acetate colorimetric method. [30] In this method, the concentration of the enzymatic product, indigo blue, was determined via UV−vis spectroscopy at a wavelength of 620 nm, with the molar extinction coefficient (ϵ) set at 22,140 $M^{-1}cm^{-1}$. This approach was chosen over the traditional Ellman method due to the susceptibility of acetylthiocholine to nucleophiles, such as oximes and imidazoles, which can result in false-positive readings.

S4.1. AChE inhibition

AChE inhibition experiments were carried out to determine diisopropylfluorophosphate ability to inhibit AChE activity at different concentrations. In a typical experiment, a 24-well culture plates were filled with 825 µL of Tris−HCl (0.1 M, pH = 7.4) buffer solution, 25 µL of an AChE aqueous solution (75 U/mL) and 100 µL of DIFP aqueous solution (DIFP final concentrations: $1·10^{-2}$ M, $1·10^{-3}$ M, $1·10^{-4}$ M, $1·10^{-5}$ M, $5·10^{-6}$ M, $1·10^{-6}$ M, $5·10^{-7}$ M, $1·10^{-7}$ M, $5·10^{-8}$ M and $1·10^{-8}$ M); and they were incubated during 1 h at 37 ºC. Then, 50 μL of indoxyl acetate solution in isopropanol (3.0 mM) was added and the mixtures were incubated for an additional 30 min. Finally, 3.33 mL of dimethylsulfoxide (DMSO) was added to solubilize the enzymatic product (indigo blue) and stop the reaction. The absorbance of the solutions was measured at λ = 620 nm.

S4.2. AChE reactivation by 2-methylimidazole

AChE reactivation experiments were carried out to determine 2-methylimidazole ability to reactivate DIFP inhibited AChE activity. In a typical experiment, a 24-well culture plates were filled with 725 µL of Tris−HCl (0.1 M, pH = 7.4) buffer solution, 25 µL of an AChE aqueous solution (75 U/mL) and 100 µL of DIFP aqueous solution $5·10^{-5}$ M (final DIFP concentration $5·10^{-6}$ M, corresponding to 50% inhibited AChE); and they were incubated during 1 h at 37 ºC. 30 minutes after DIFP addition, we added 100 µL of mImH aqueous solution (mImH final concentrations: $1·10^{-1}$ M, $1·10^{-2}$ M, $1·10^{-3}$ M, $1·10^{-4}$ M, $1·10^{-5}$ M, $1·10^{-6}$ M, $1·10^{-7}$ M and $1·10^{-8}$ M). 1 h after DIFP addition, 50 μL of indoxyl acetate solution in isopropanol (3.0 mM) was added and the mixtures were incubated for an additional 30 min. Finally, 3.33 mL of dimethylsulfoxide (DMSO) was added to solubilize the enzymatic product (indigo blue) and stop the reaction. The absorbance of the solutions was measured at λ = 620 nm.

S4.3. Detoxification experiments

Detoxification experiments were carried out to study the ability of synthesized ZIF-L(_exf) materials to reduce DIFP inhibition activity towards AChE. To establish differences between both materials, the detoxification process was followed at different incubation times (10 min, 30 min, 1 h, 3 h and 24 h). We employed the same experimental conditions as in the catalytic assays in Tris-HCl buffered solution. In this case, 0.084 mmol of ZIF-L(_exf) was combined with DIFP (2.5 µL, 0.015 mmol) in 0.5 mL of Tris−HCl buffer solution (0.1 M, pH = 7.4) and incubated at room temperature. After different time frames, the supernatant was separated by centrifugation (15,000 rpm x 5 min) and diluted 580 times. Afterwards, 24-well culture plates were filled with 825 µL of Tris−HCl (0.1 M, pH = 7.4) buffer solution, 25 µL of an AChE aqueous solution (75 U/mL) and 100 µL of previously diluted supernatants; and they were incubated during 1 h at 37 ºC. Then, 50 μL of indoxyl acetate solution in isopropanol (3.0 mM) was added and the mixtures were incubated for an additional 30 min. Finally, 3.33 mL of dimethylsulfoxide (DMSO) was

added to solubilize the enzymatic product (indigo blue) and stop the reaction. The absorbance of the solutions was measured at λ = 620 nm.

In addition, control experiments were performed to determine the enzymatic activity of uninhibited and 50 % inhibited AChE. For the uninhibited AChE experiments, the culture plate was filled with 925 µL of Tris−HCl (0.1 M, pH = 7.4) buffer solution and 25 µL of an AChE aqueous solution (75 U/mL). For the 50 % inhibited AChE experiments, the culture plate was filled with 825 µL of Tris−HCl (0.1 M, pH = 7.4) buffer solution, 25 µL of an AChE aqueous solution (75 U/mL) and 100 µL of DIFP aqueous solution $5 \cdot 10^{-5}$ M (DIFP final concentration is $5 \cdot 10^{-6}$ M, corresponding to 50 % AChE inhibition). Both control experiments were incubated 1 h at 37 ºC. Then, 50 μL of indoxyl acetate solution in isopropanol (3.0 mM) was added and the mixtures were incubated for an additional 30 min. Finally, 3.33 mL of dimethylsulfoxide (DMSO) was added to solubilize the enzymatic product (indigo blue) and stop the reaction. The absorbance of the solutions was measured at λ = 620 nm.

The detoxification percentage value was calculated according to the following expression:

$$\text{Detoxification (\%)} = ((A_{ZIF}-A_{INH})/A_{INH})*100$$

Where,

A_{ZIF} = AChE activity for ZIF-L(_exf) detoxificacion experiments

A_{INH} = AChE activity for 50% ihibited control experiments

S5. G-type nerve agent simulant degradation with Dose-Extraction method

DIFP degradation by ZIF-L(_exf) materials was also studied using a dose-extraction method. 30 mg of powder was placed in a small vial and equilibrated at 50% RH overnight. Then, 2.5 μL (10 wt% with respect to ZIF-L) of DIFP was pipetted onto the powder, and then the vial was vortexed to ensure maximum dispersion. Then, the powders were extracted with ~1 mL of acetonitrile. The solvent was passed through a nylon syringe filter, and the concentration of DIFP in the extract was determined by GC-FID.

S6. Real nerve agent degradation with Dose-Extraction method

ZIF-L and ZIF-L_exf materials were testing for reactivity towards O-pinacolyl methylphosphonofluoridate, aka Soman (GD) using a dose-extraction method. Briefly, ~30 mg of powder was placed in a small vial and equilibrated at 50% RH overnight. Then, 3 µL (10 wt% with respect to ZIF-L) of neat GD was pipetted onto the powder, and then the vial was vortexed to ensure maximum dispersion. After 1, 4, and 24 h, the vials were cooled in a dry ice bath to halt the reaction, and then the powders were extracted with ~1 mL of acetonitrile. The solvent was passed through a nylon syringe filter, and the concentration of GD in the extract was determined by an Agilent 6890 gas chromatograph equipped with a 5973 mass spectrometer.

S7. DFT calculations

All DFT calculations were performed in the Gaussian 09,[31] using the TPSS (meta-GGA)[32] functional with def2-TZV[33, 34] on all atoms and SMD[35] implicit solvation model using water as the solvent. Geometry optimizations were

computed in solution without symmetry restrictions. All calculated structures were stationary points as confirmed by single-point vibrational frequency calculations. Free energy corrections were calculated at 298.15 K and 105 Pa pressure, including zero-point energy corrections (ZPE). Unless otherwise mentioned, all reported energy values are free energies in solution under standard state conditions. The DFT calculations with the optimized xyz coordinates are available in the ioChem-BD database.[36]

S8. Figures

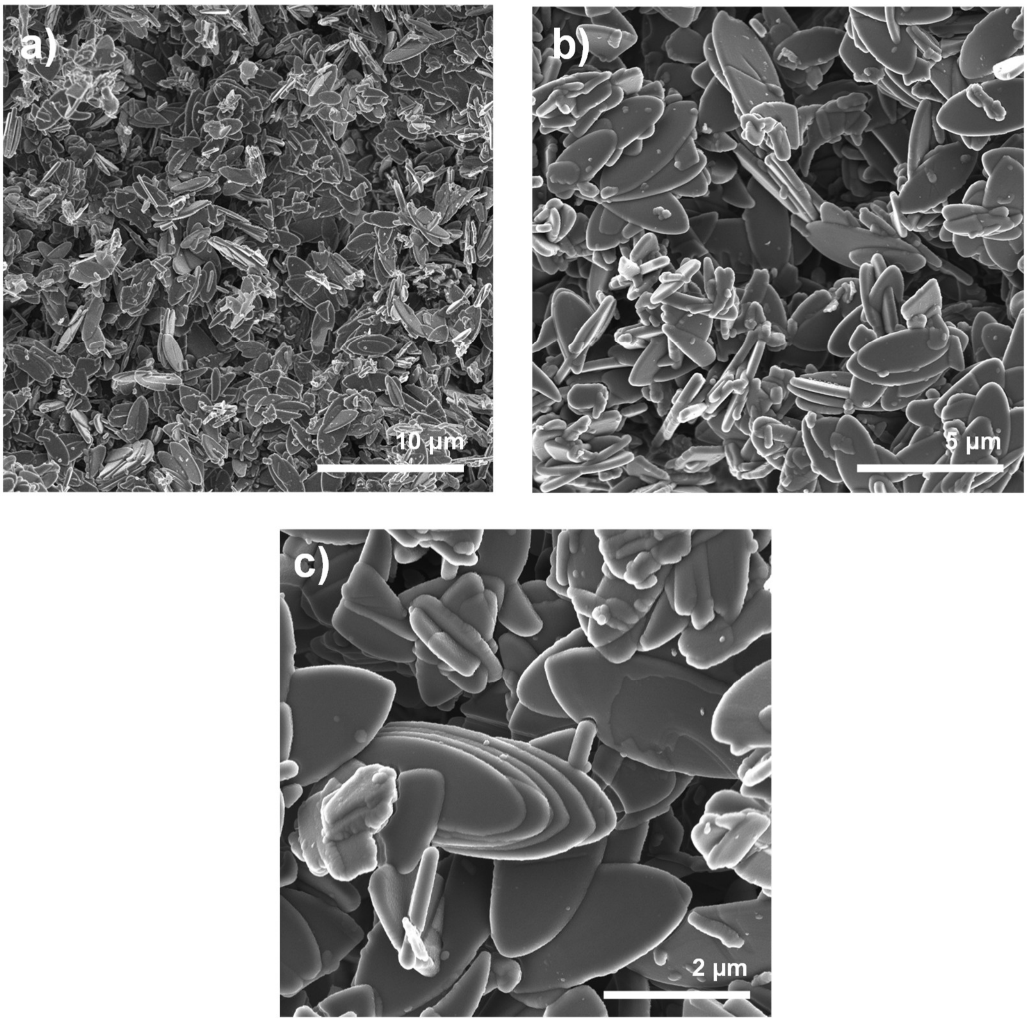

Figure S1. Scanning Electron Microscopy images of **ZIF-L** at different magnifications: a) 10 µm, b) 5 µm and c) 2 µm.

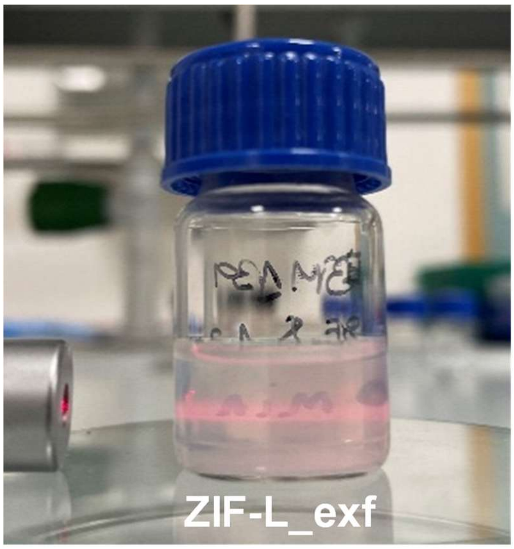

Figure S2. Tyndall effect observed in **ZIF-L_exf** suspension in EtOH

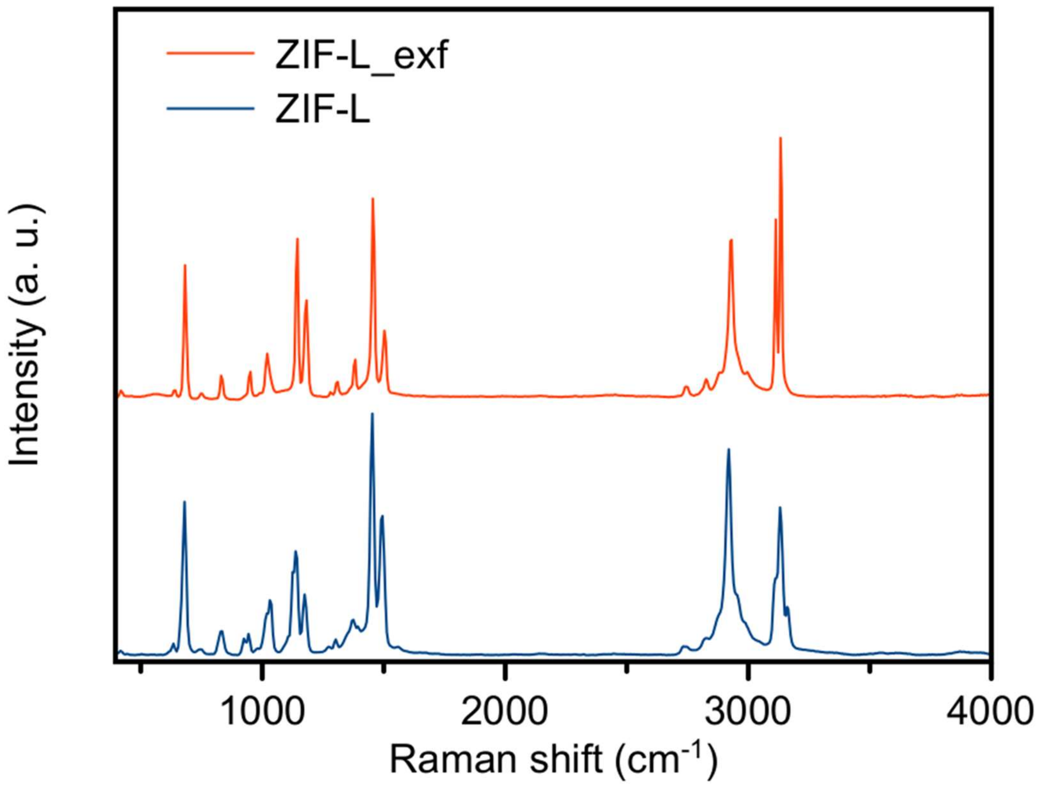

Figure S3. RAMAN spectra of pristine **ZIF-L** (blue) and **ZIF-L exf** (red). **ZIF-L** powder was directly placed over laboratory glass plates. **ZIF-L_exf** was analysed by depositing 30 μL of the sonicated suspension on the glass plate and allowing it to air dry.

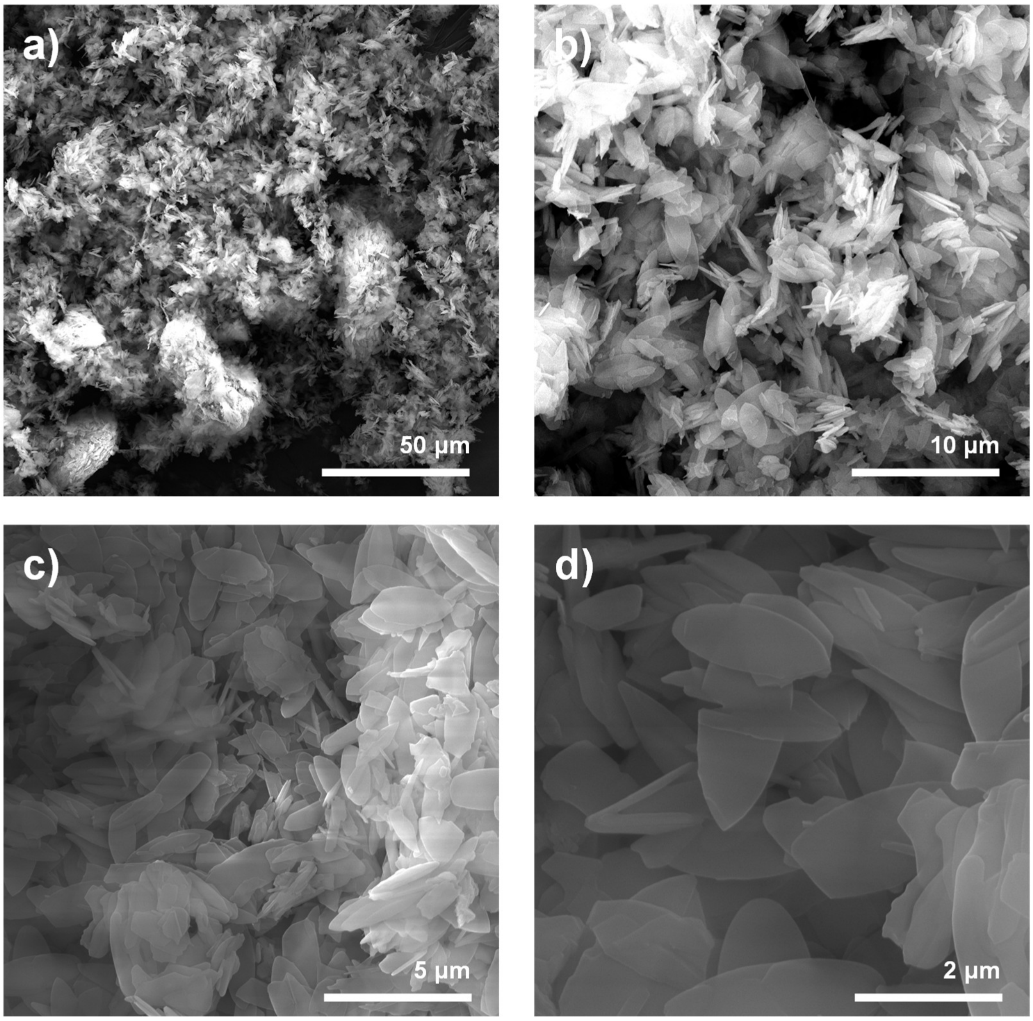

Figure S4. Scanning Electron Microscopy images of **ZIF-L exfoliated** in EtOH during 5 min at different magnifications: a) 50 µm, b) 10 µm, c) 5 µm and d) 2 µm.

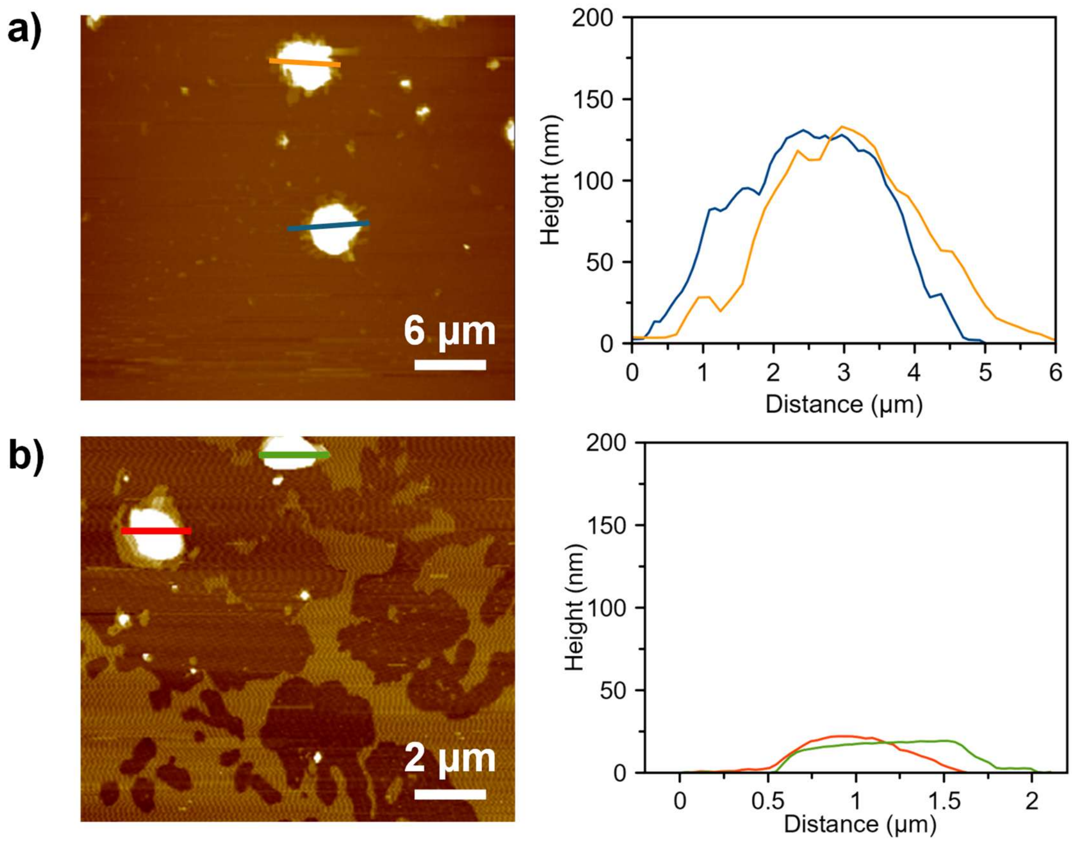

Figure S5. Atomic Force Microscopy images of **(**a) **ZIF-L** and (b) **ZIF-L_exf** materials. Both materials were dropwise deposited onto mica sample holder previously cleaved using a sticky tape. **ZIF-L** was suspended in EtOH (0.1 mg·mL^{-1}), sonicated during 1 minute and 30 µL of this suspension was dropped onto mica by using a spin-coater (250 rpm, 2 min.). **ZIF-L_exf** sample was prepared by diluting 2 times the previously exfoliated suspension (see S2.2.) and suspending by drop casting onto mica sample holder using a spin-coater (300 rpm, 3 min.).

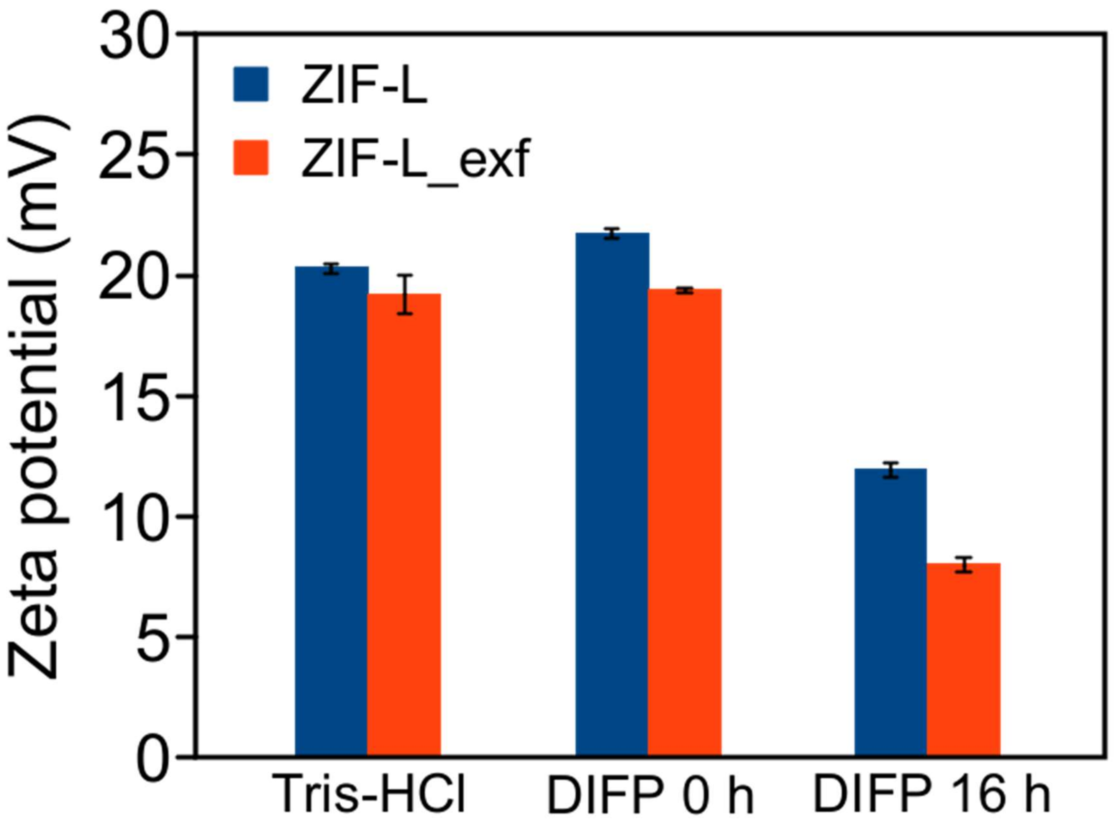

Figure S6. Z potential measurements of **ZIF-L(_exf)** suspension after DIFP hydrolytic degradation process in Tris-HCl (0.1 M, pH = 7.4).

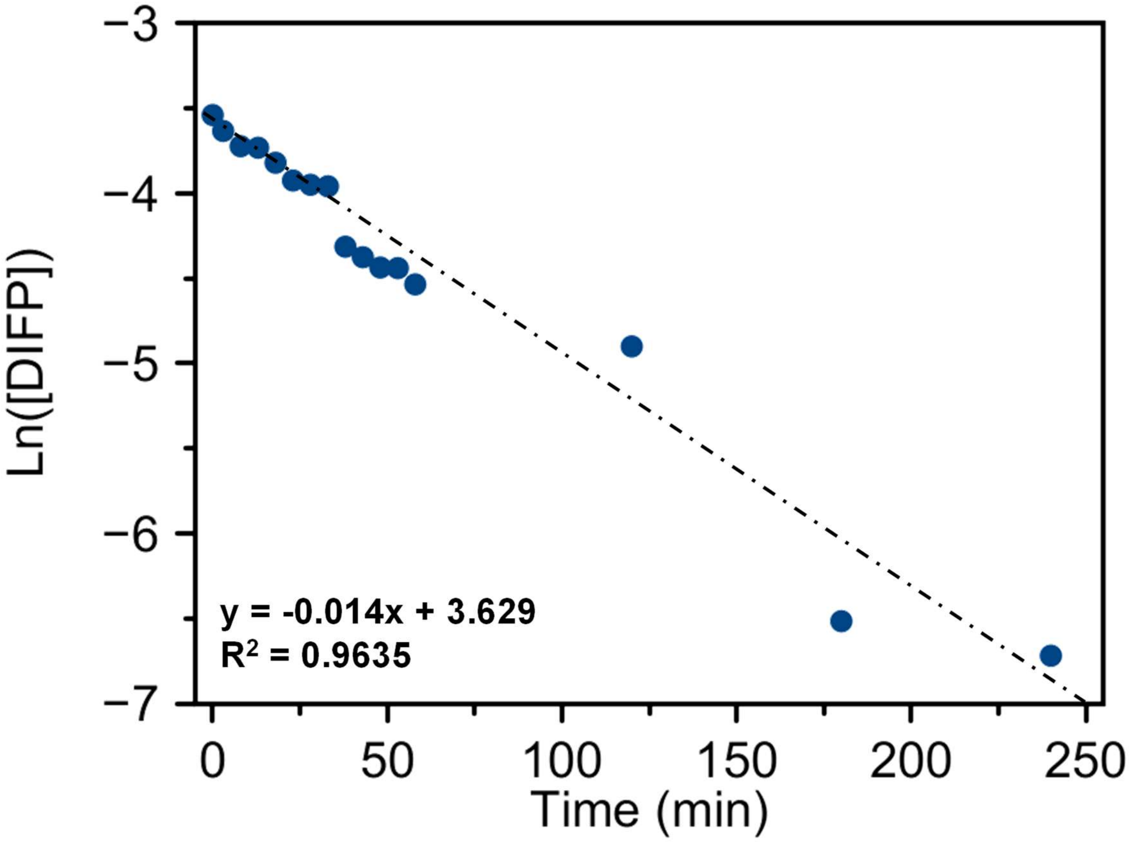

Figure S7. Fitting curve to first-order kinetic model of DIFP hydrolytic degradation by **ZIF-L** in Tri-HCl (pH = 7.4, 0.1 M) buffer suspension.

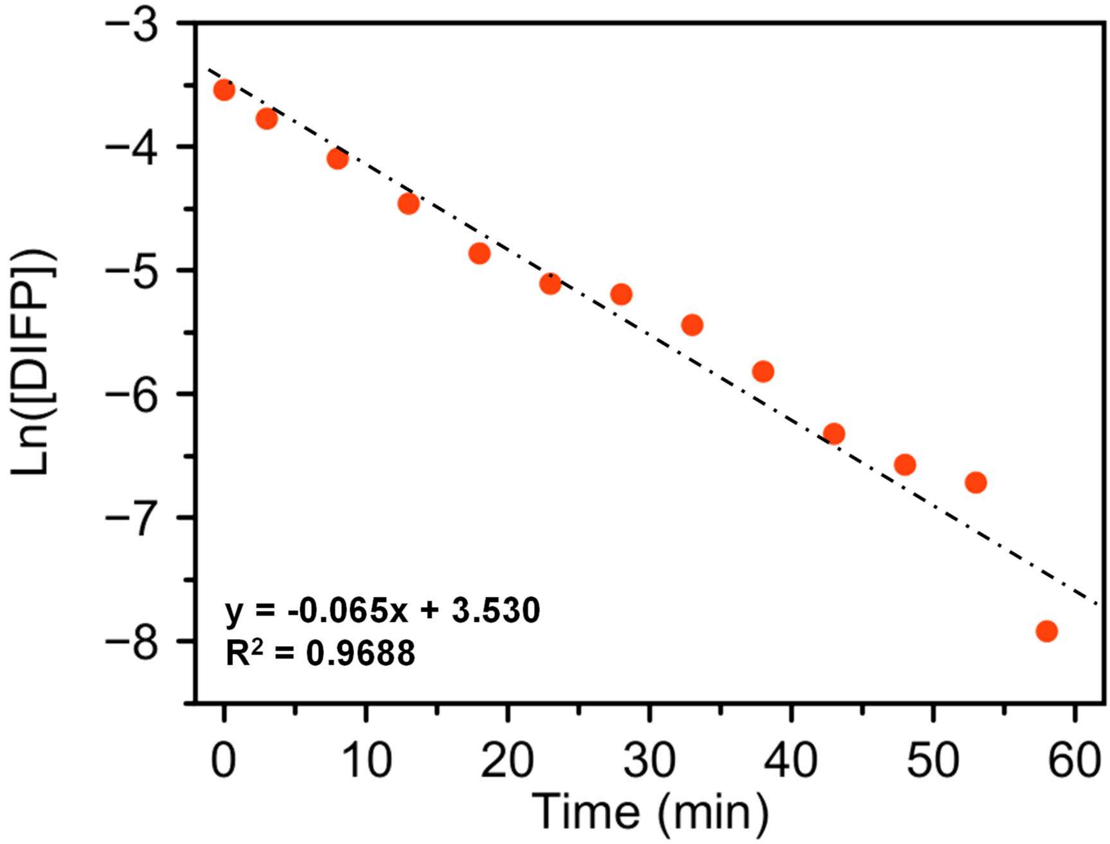

Figure S8. Fitting curve to first-order kinetic model of DIFP hydrolytic degradation by **ZIF-L_exf** in Tri-HCl (pH = 7.4, 0.1 M) buffer suspension.

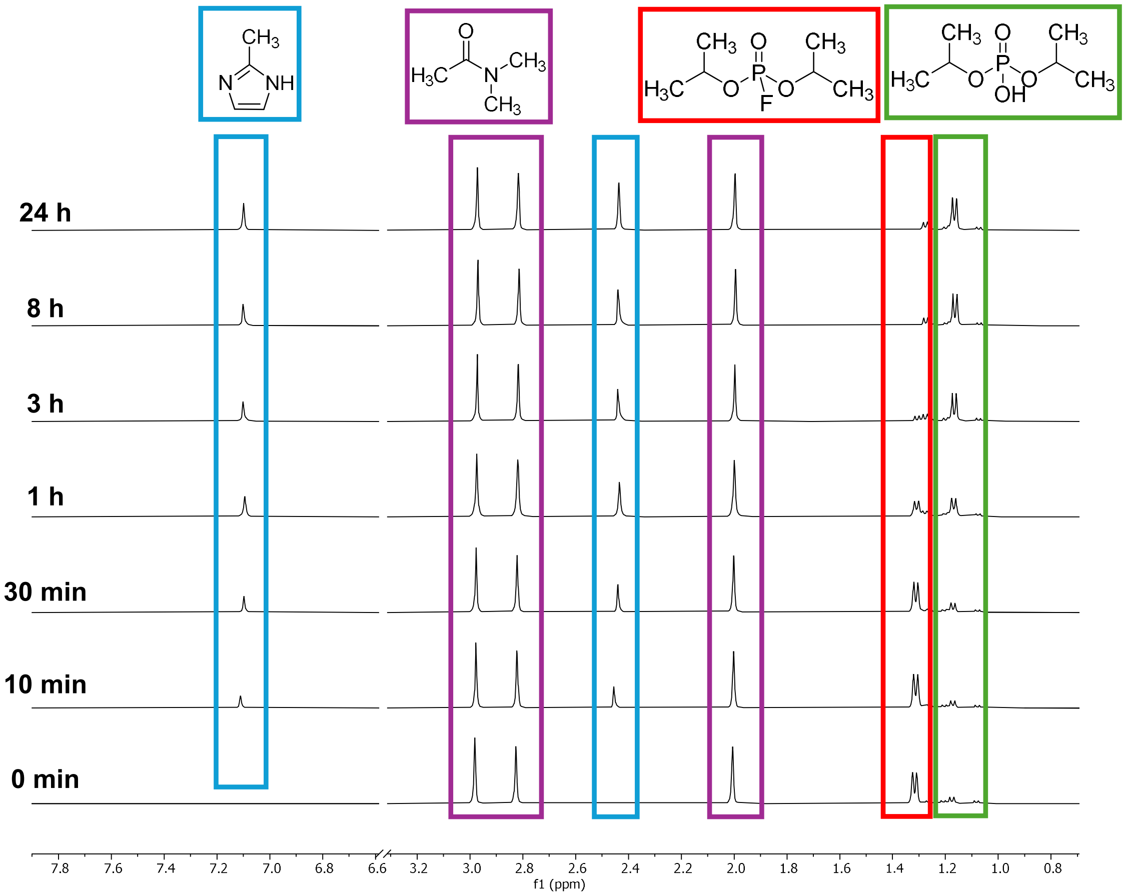

Figure S9. ^{1}H-NMR of **ZIF-L** suspension during DIFP hydrolytic degradation to study 2-methylimidazole release. Experimental conditions: 0.5 mL Tris-DCl (0.1 M, pD = 7.0), 0.015 mmol DIFP, 0.015 mmol Dimethylacetamide (DMA, internal standard) and 0.029 mmol **ZIF-L**.

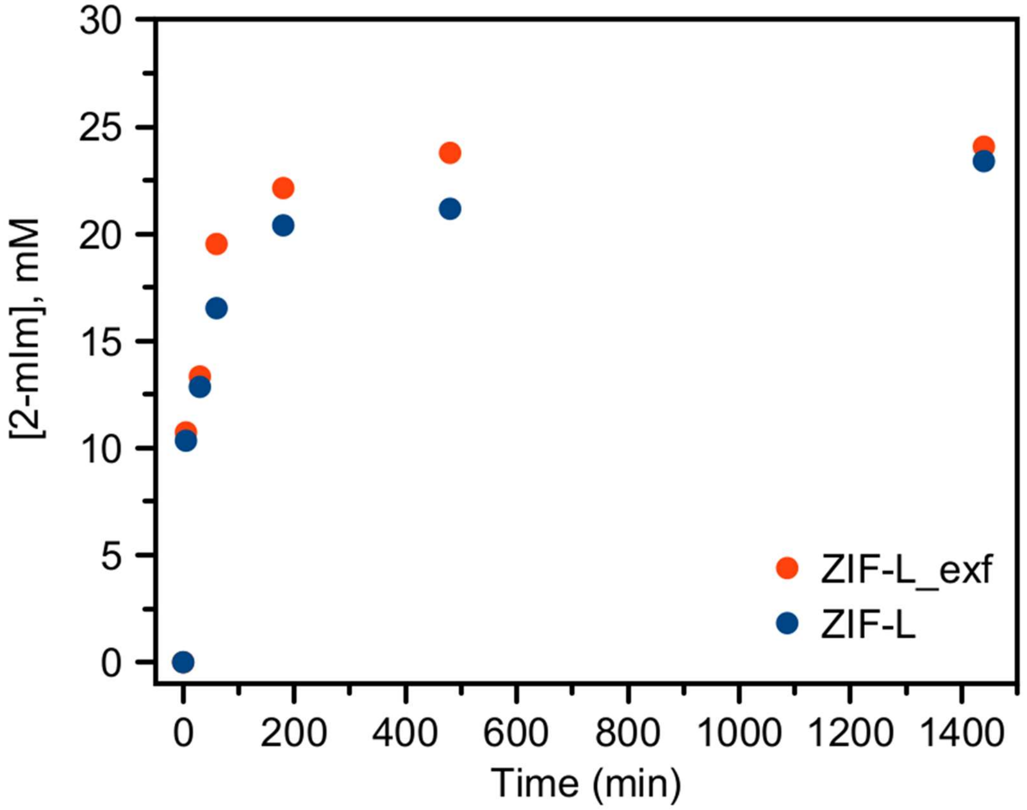

Figure S10. 2-methylimidazole linker release profile from **ZIF-L(_exf)** suspension in DIFP hydrolytic degradation.

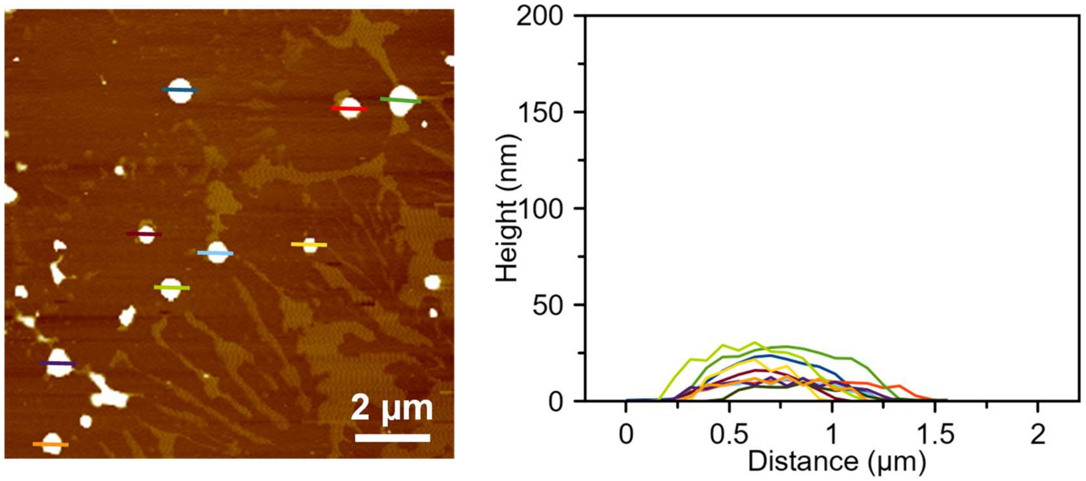

Figure S11. Atomic Force Microscopy images of **ZIF-L_exf** material after DIFP degradation process. After DIFP reaction ZIF-L_exf suspension was washed with EtOH by triplicate (15,000 rpm x 5min) and suspended in EtOH (0.1 mg·mL^{-1}). 30 µL of this suspension was dropwise deposited onto mica sample holder (previously cleaved using a sticky tape) by using a spin-coater (250 rpm, 2 min.).

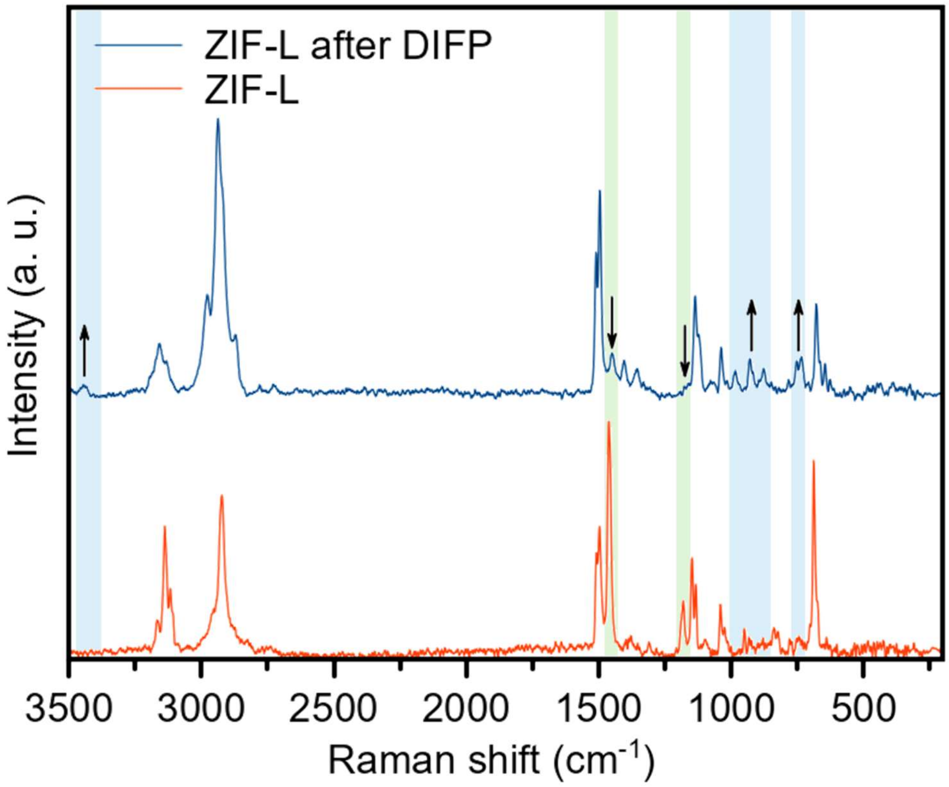

Figure S12. Fourier Transformed Raman spectra of **ZIF-L** crystal surface before (red) and after (blue) exposition to **DIFP** Tris-HCl buffer (see section S3.1.).

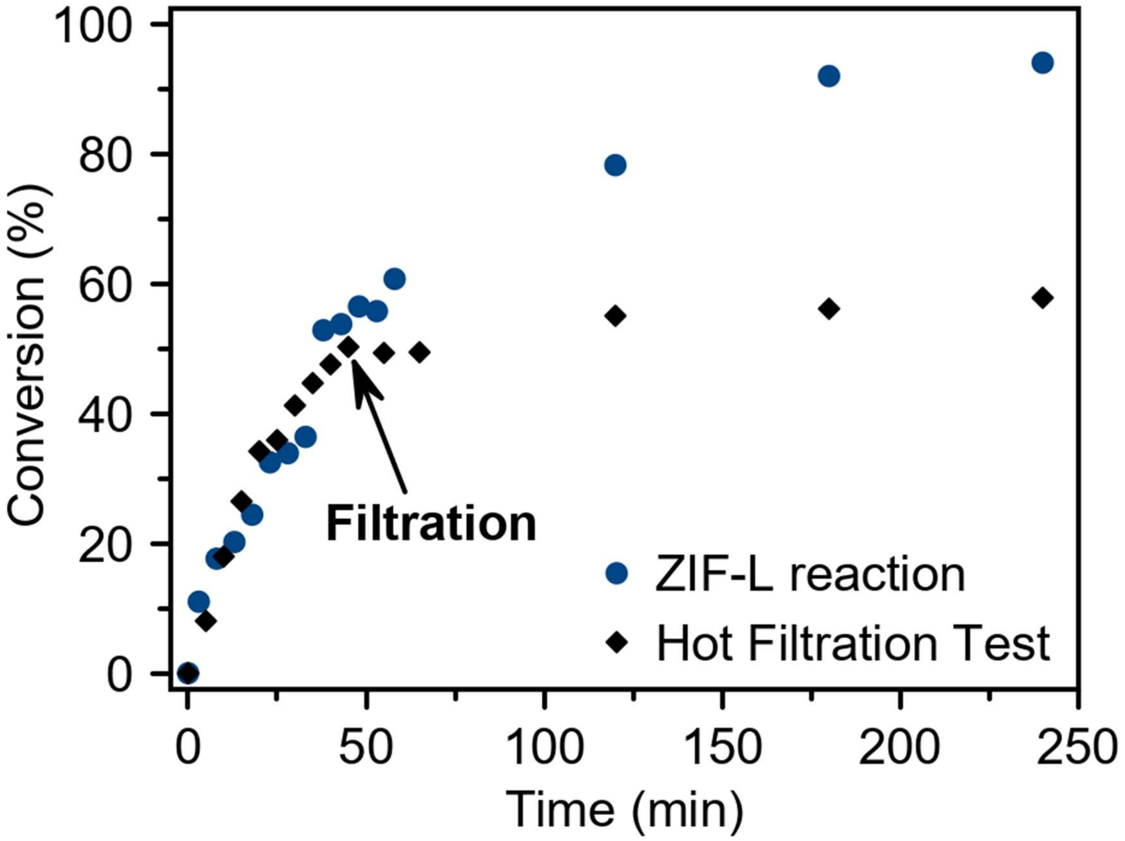

Figure S13. Hot filtration test for diisopropylfluorophosphate degradation process by **ZIF-L** in Tris-HCl buffer solution (0.1 M, pH = 7.4). DIFP degradation was followed by GC-FID.

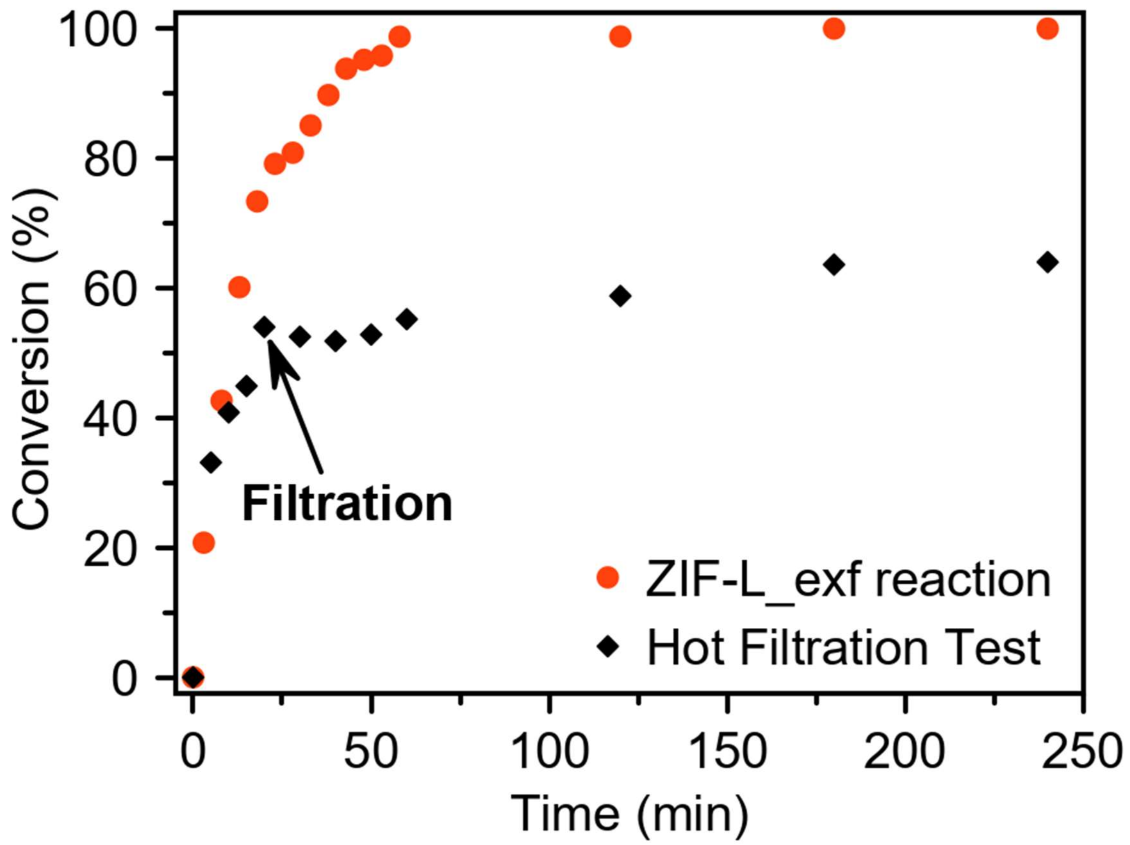

Figure S14. Hot filtration test for diisopropylfluorophosphate degradation process by **ZIF-L_exf** in Tris-HCl buffer solution (0.1 M, pH = 7.4). DIFP degradation was followed by GC-FID.

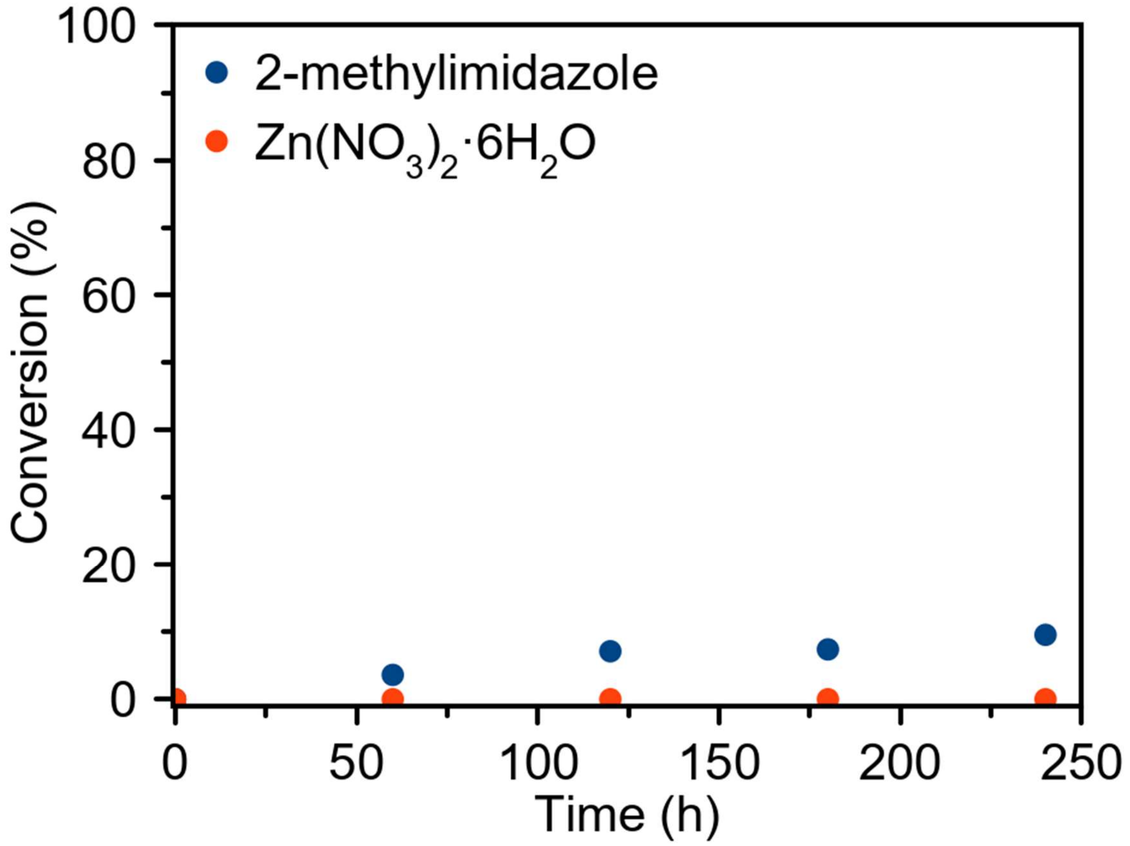

Figure S15. Profile of diisopropylfluorophosphate (0.015 mmol) degradation process by $Zn(NO_3)_2 \cdot 6H_2O$ and 2-methylimidazole precursors in Tris-HCl buffer solution (0.1 M, pH = 7.4). Metal and ligand concentrations were selected according to **ZIF-L** release of ligand after 24 h. DIFP degradation was followed by GC-FID.

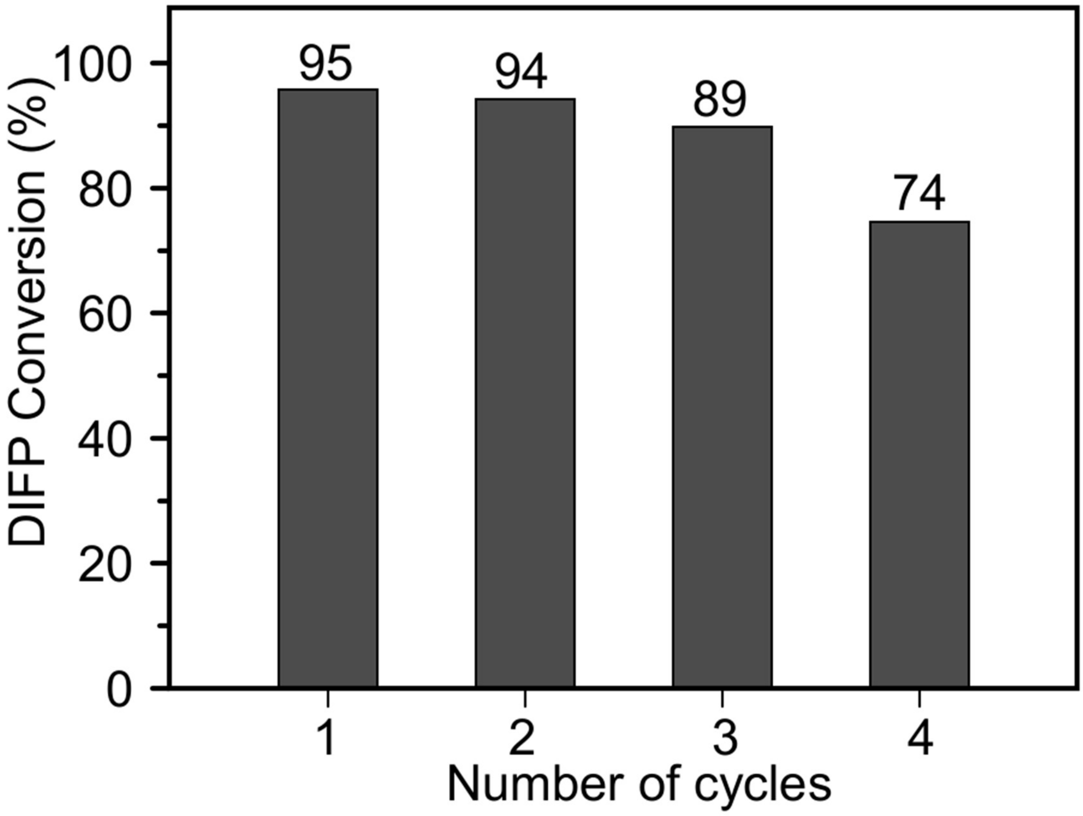

Figure S16. Recyclability test for diisopropylfluorophosphate degradation process by **ZIF-L** (25 mg, 0.0845 mmol) in Tris-HCl buffer solution (0.1 M, pH = 7.4). A pulse of DIFP (2.5 μL, 0.015 mmol) was added every 4 hours and degradation was followed by GC-FID. Between each cycle, the material was washed with water.

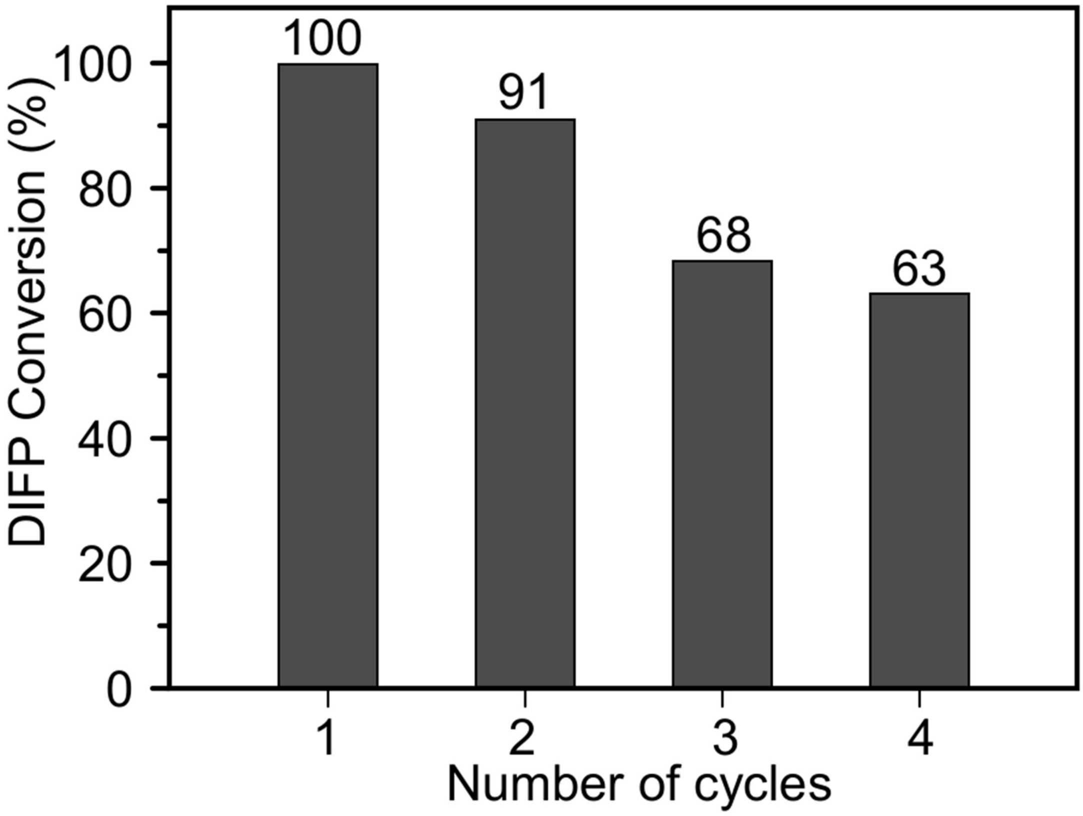

Figure S17. Recyclability test for diisopropylfluorophosphate degradation process by **ZIF-L_exf** (25 mg, 0.0845 mmol) in Tris-HCl buffer solution (0.1 M, pH = 7.4). A pulse of DIFP (2.5 μL, 0.015 mmol) was added every 4 hours and degradation was followed by GC-FID. Between each cycle, the material was washed with water.

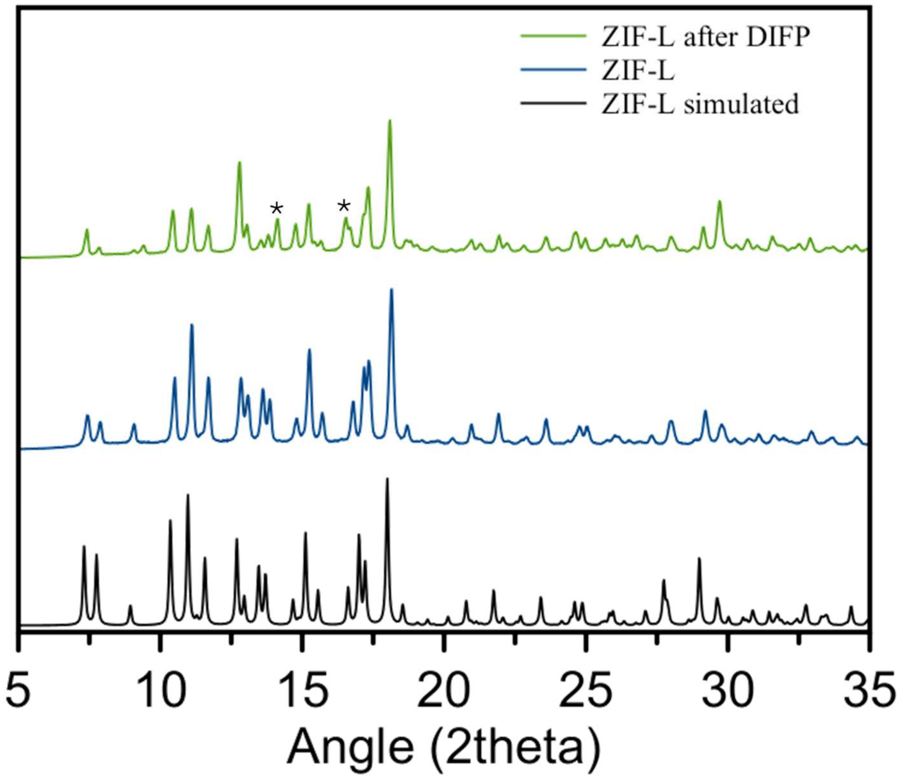

Figure S18. Powder X-Ray Diffraction of pristine **ZIF-L** and **ZIF-L after DIFP** hydrolytic degradation in Tris-HCl buffer (see S3.1.). New crystalline phase appearance denoted with asterisks.

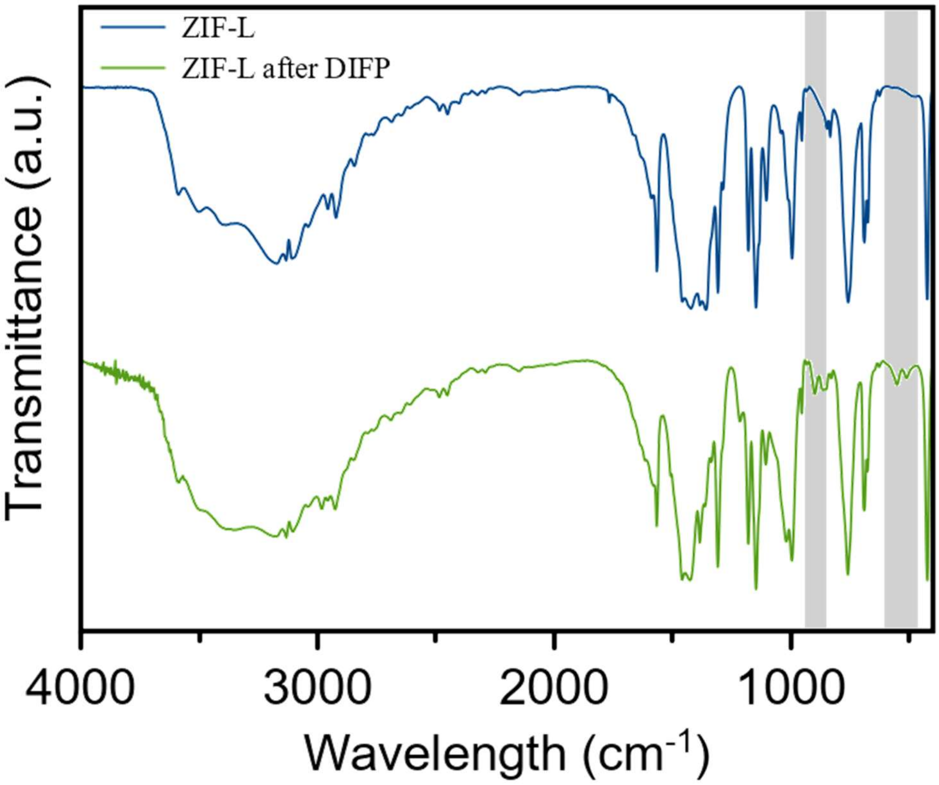

Figure S19. Fourier Transformed Infrared spectra of pristine **ZIF-L** and **ZIF-L after DIFP** hydrolytic degradation in Tris-HCl buffer (see S3.1.).

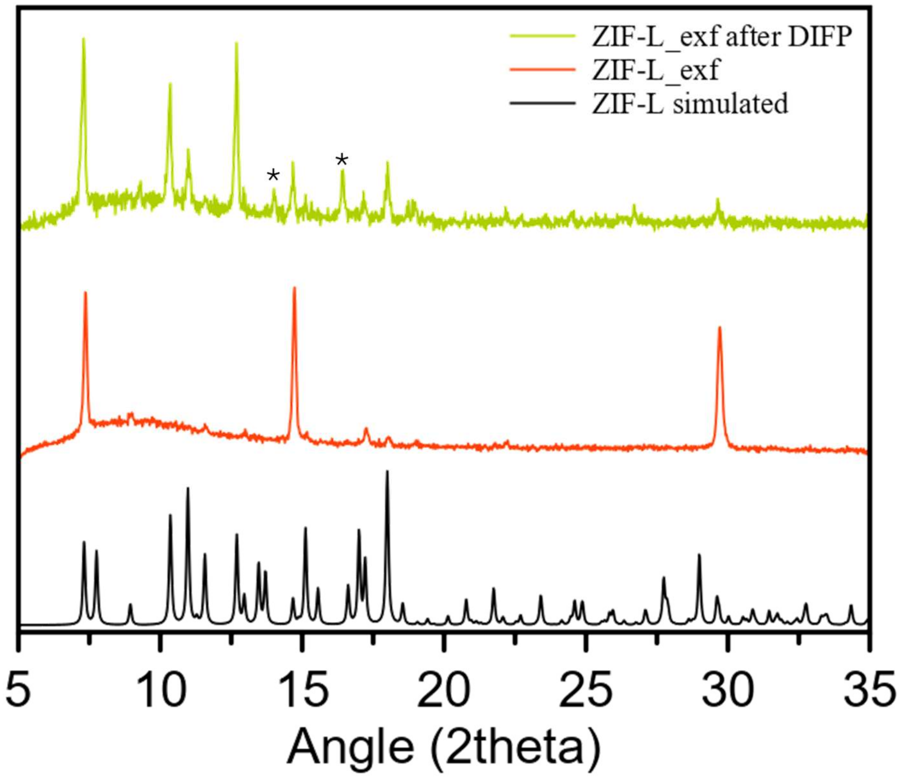

Figure S20. Powder X-Ray Diffraction of **ZIF-L_exf** and **ZIF-L_exf after DIFP** hydrolytic degradation in Tris-HCl buffer (see S3.1.). New crystalline phase appearance denoted with asterisks.

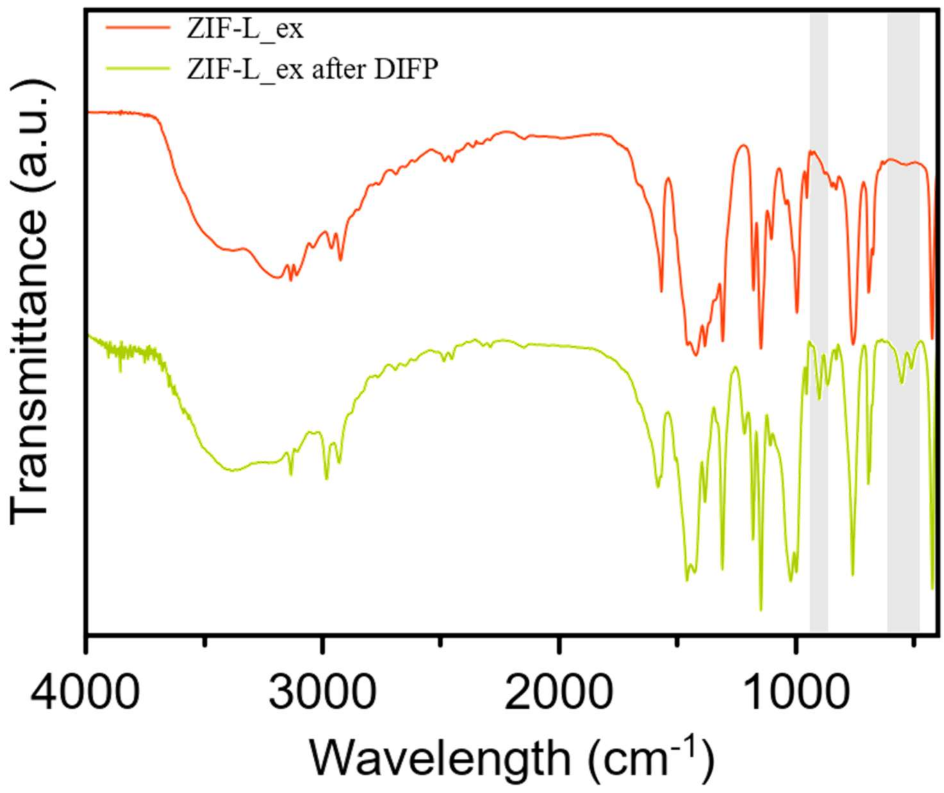

Figure S21. Fourier Transformed Infrared spectra of pristine **ZIF-L_exf** and **ZIF-L_exf after DIFP** hydrolytic degradation in Tris-HCl buffer (see S3.1.).

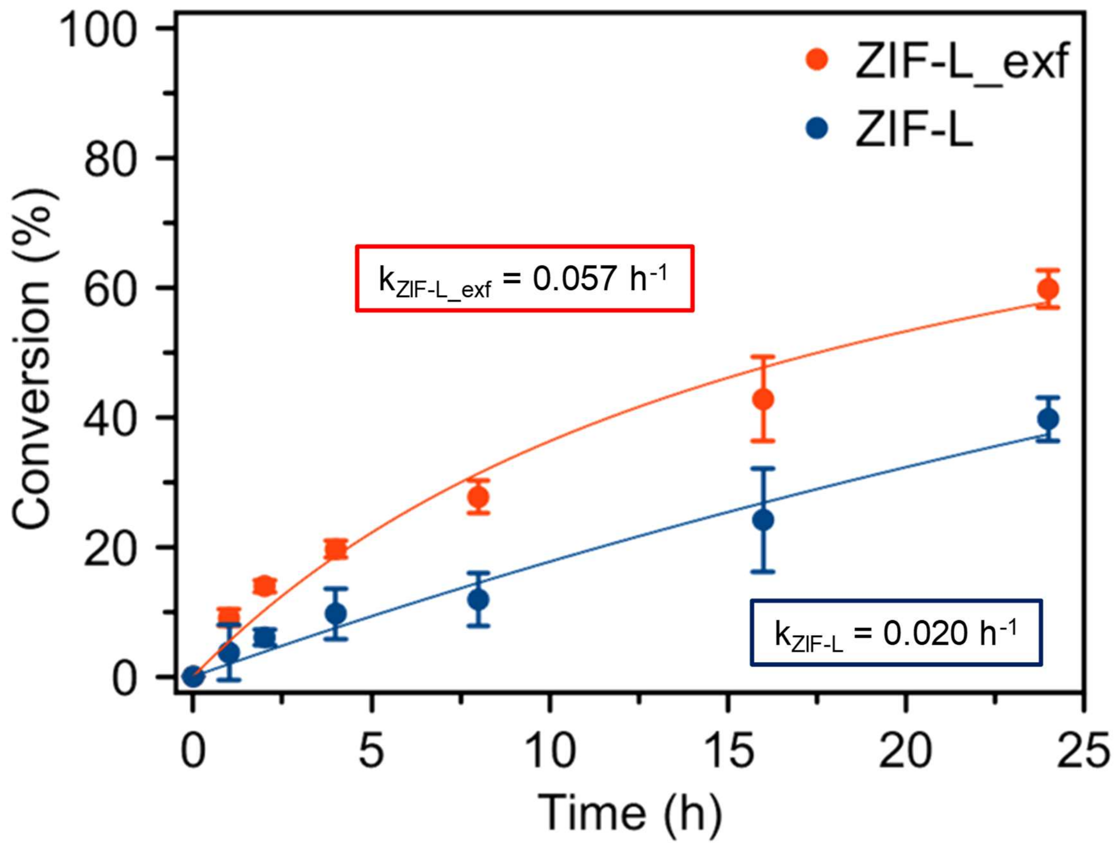

Figure S22. Profile of diisopropylfluorophosphate (0.015 mmol) hydrolytic degradation process by **ZIF-L(_exf)** materials using the dose-extraction method with 1 mL of acetonitrile. DIFP degradation was followed by GC-FID.

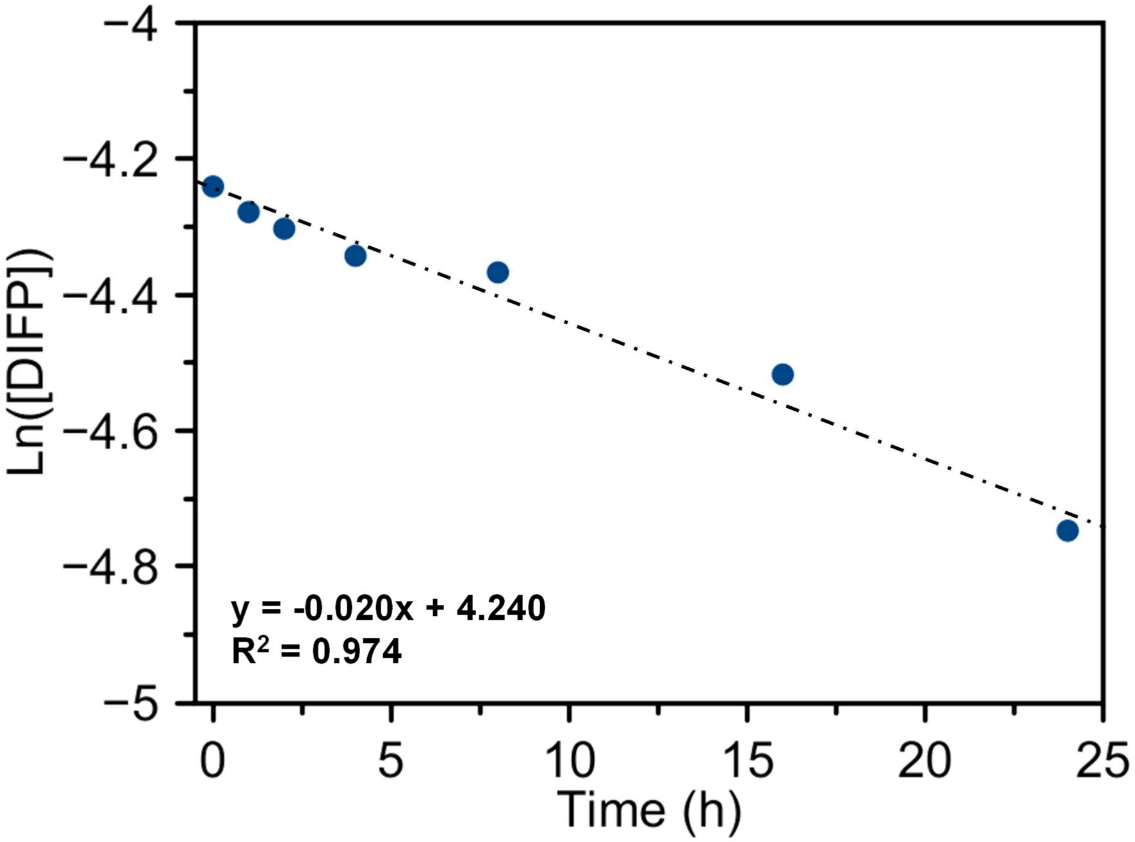

Figure S23. Fitting curve to first-order kinetic model of DIFP hydrolytic degradation by **ZIF-L** using the dose-extraction method.

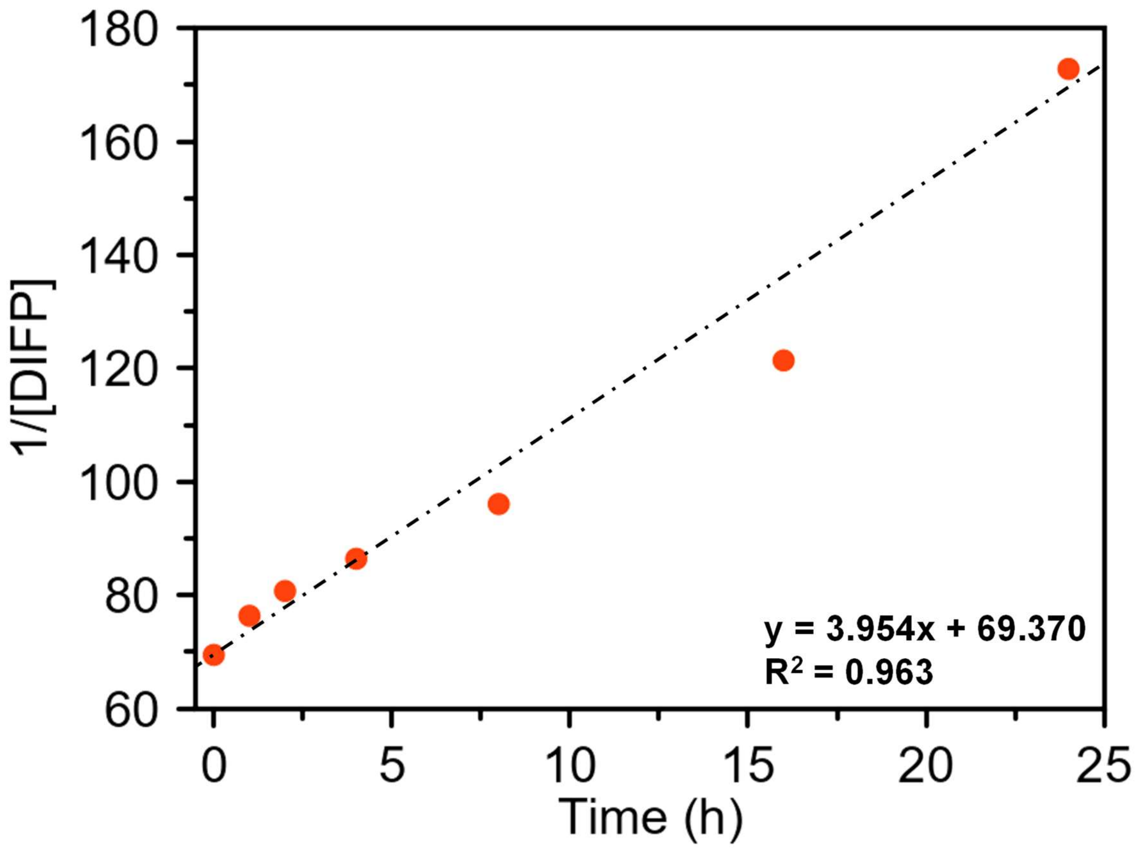

Figure S24. Fitting curve to second-order kinetic model of DIFP hydrolytic degradation by **ZIF-L_exf** using the dose-extraction method.

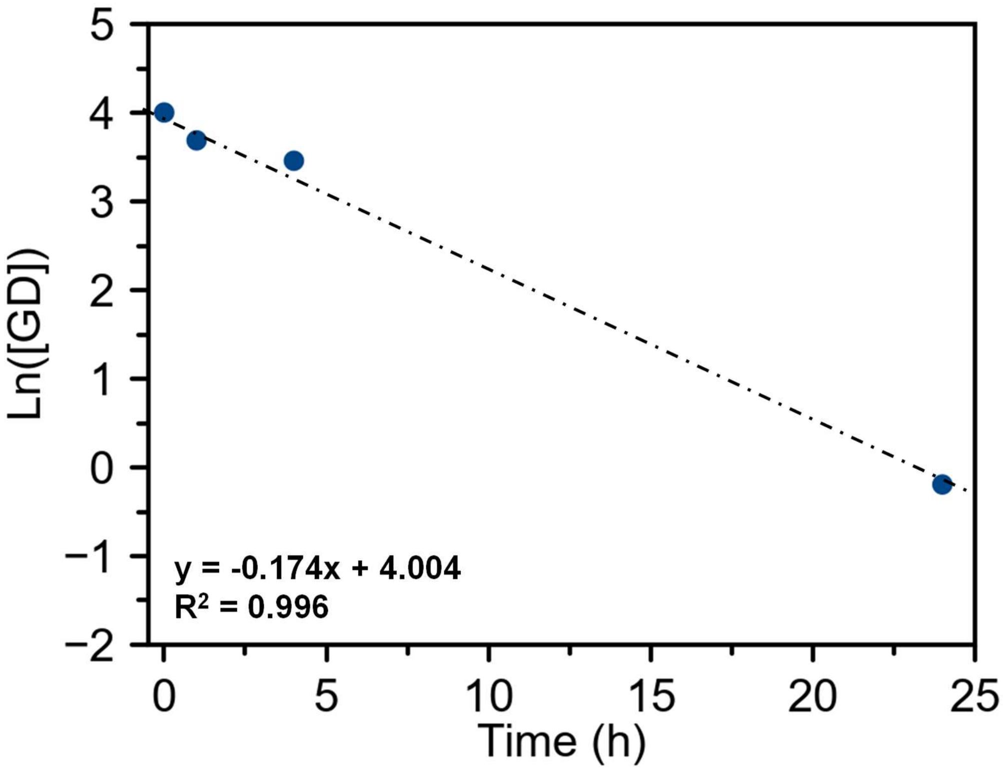

Figure S25. Fitting curve to first-order kinetic model of Soman hydrolytic degradation by **ZIF-L** using the dose-extraction method.

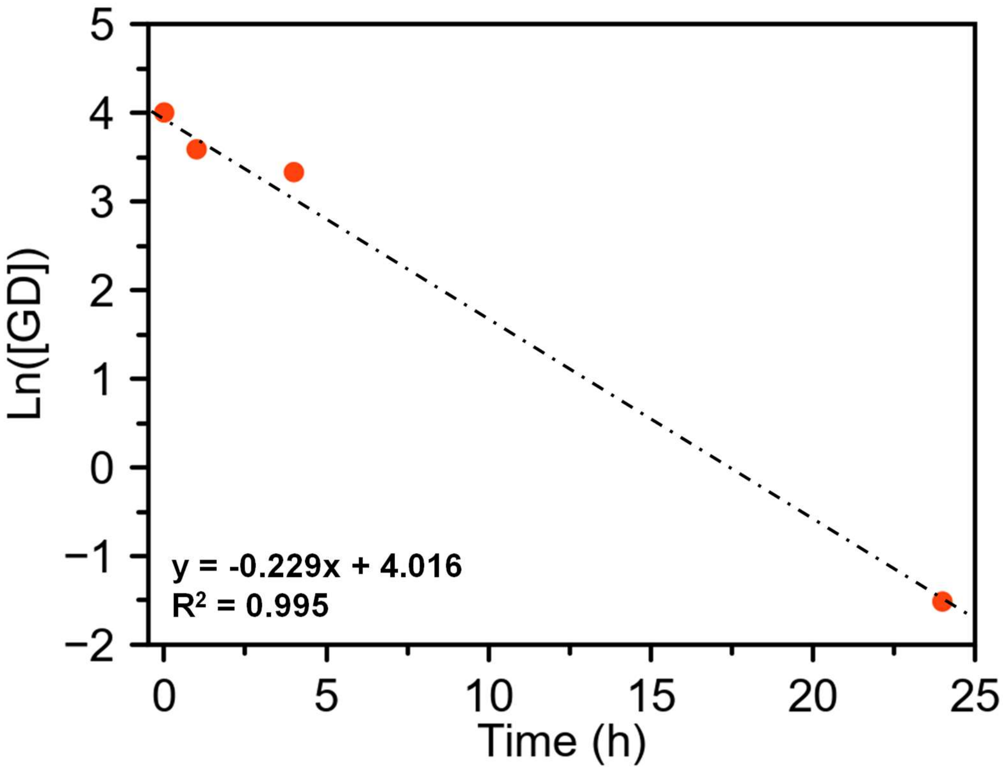

Figure S26. Fitting curve to first-order kinetic model of Soman hydrolytic degradation by **ZIF-L** using the dose-extraction method.

a)

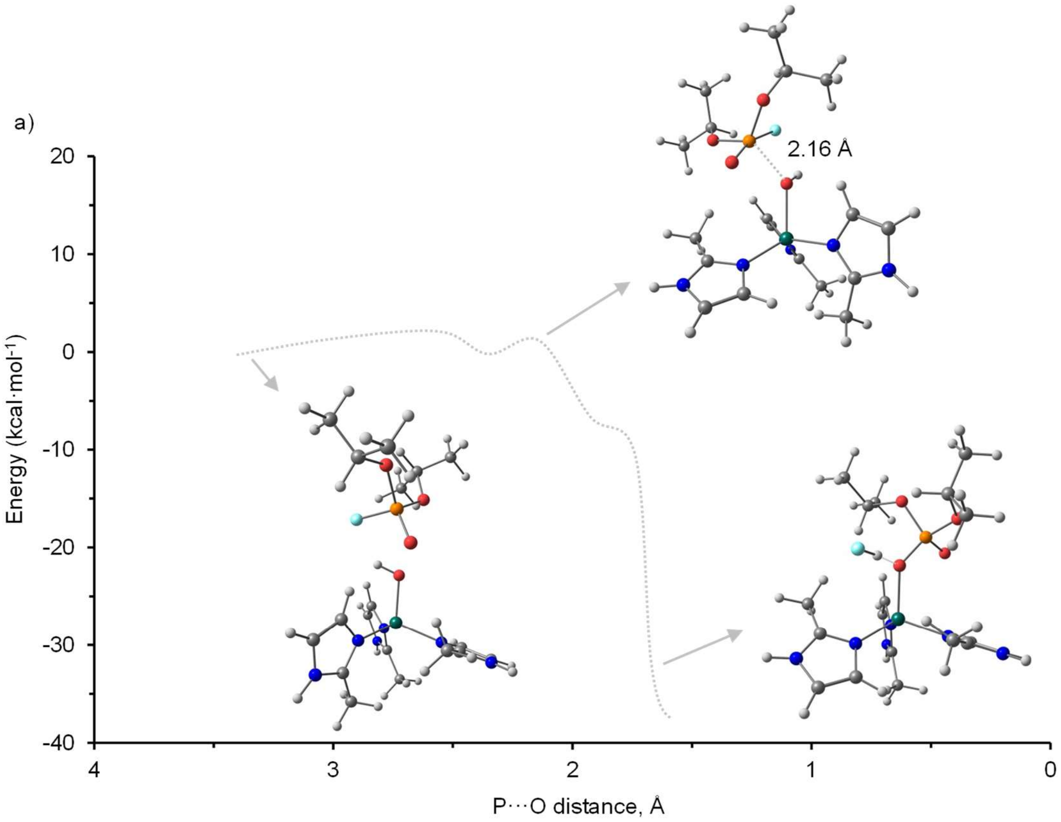

b)

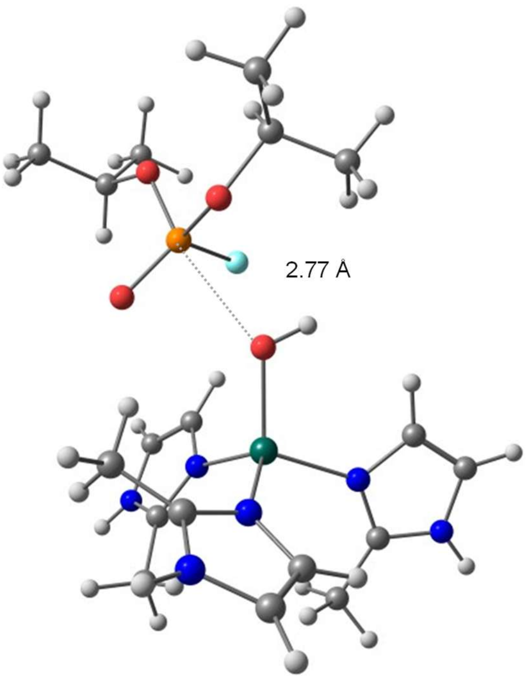

Figure S27. (a) Energy reaction profile for Zn-OH nucleophilic attack to P atom of DIFP to yield Zn-DIP + HF. (b) Structure of a DFT optimized transition state involving the attack of the Zn-OH to the P in the DIFP molecule.

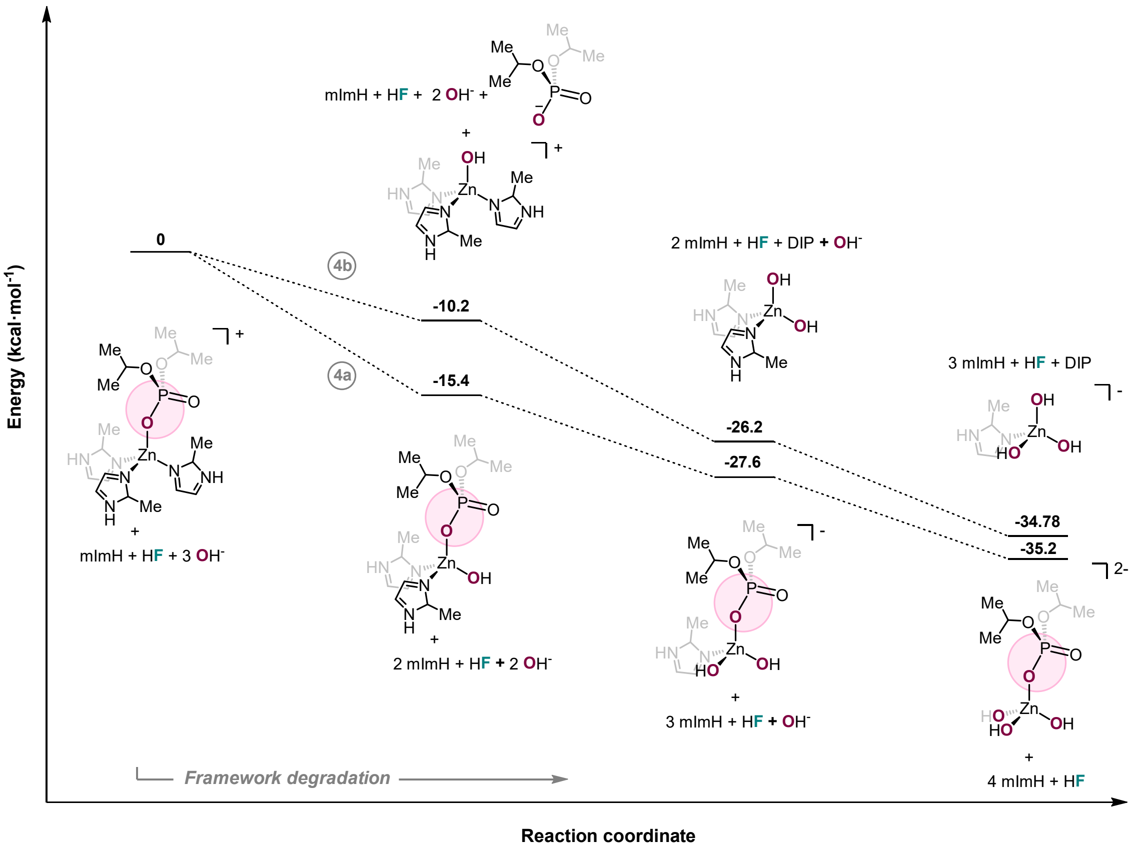

Figure S28. DFT calculations for possible reactions pathways for the outcome of DIFP reactivity towards **ZIF-L_exf** crystal surface: i) 4a pathway of hydrolytic breakdown of Zn-mIm bond leading to framework structural degradation; ii) 4b pathway of hydrolytic breakdown of Zn-DIP bond with regeneration of active center; iii-iv) successive DIFP induced surface degradation.

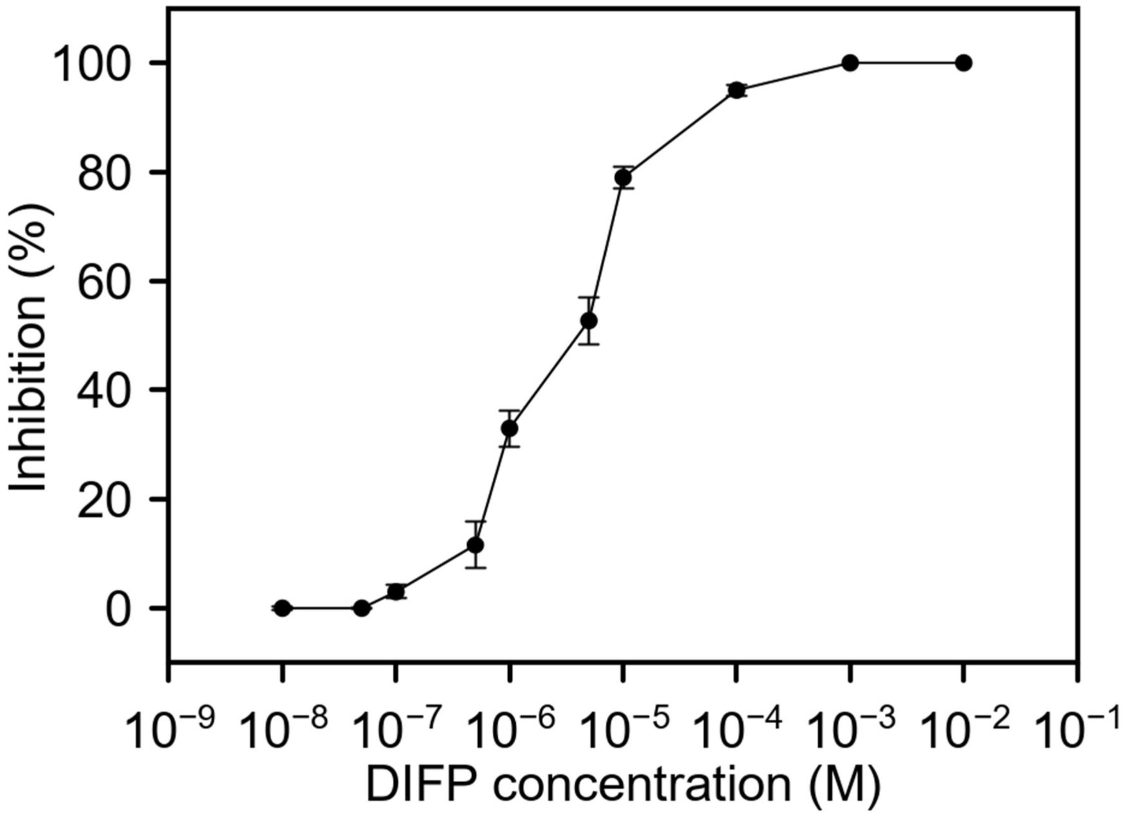

Figure S29. Profile of AChE inhibition by diisopropylfluorophosphate at different concentrations. Inhibition percentage was calculated by comparing AChE inhibited activity with free AChE.

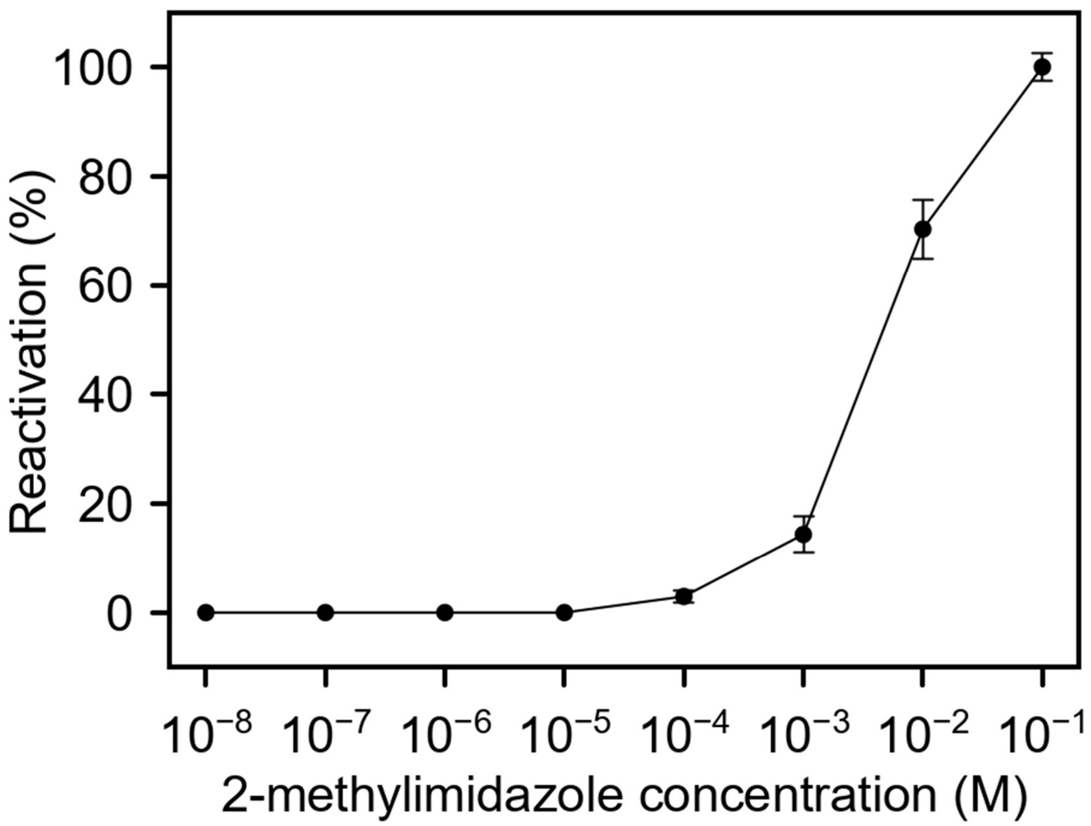

Figure S30. Reactivation profile for 50% DIFP inhibited AChE by 2-methylimidazole.

S9. Tables

Table S1. Soman degradation by **ZIF-L** and **ZIF-L_exf** materials using a dose-extraction method.

Time (h)	% Soman degradation	
	ZIF-L	ZIF-L_exf
0	0	0
1	27	34
4	42	49
24	100	100

Table S2. Released mImH concentrations upon incubation of ZIF-L(_exf) with DIFP (see S3.1.).

Time (min)	mImH concentration (mM)	
	ZIF-L	ZIF-L_exf
0	0	0
5	10.3	10.7
30	12.9	13.3
60	16.5	19.5
180	20.4	22.1
480	21.2	23.8
1440	23.4	24.1

S10. References

(29) R. Chen, J. Yao, Q. Gu, S. Smeets, C. Baerlocher, H. Gu, D. Zhu, W. Morris, O. Yaghi, Wang H. *Chem. Comm.*, **2013**, 9500-9502, 49(82).

(30) M. Pohanka, M. Hrabinova, K. Kuca, J. P. Simonato. *Int. J. Mol. Sci.* **2011**, 12 (4), 2631−2640.

(31) Gaussian 09, Revision D.01, M. J. Frisch; G. W. Trucks, H. B. Schlegel, G. E. Scuseria, M. A. Robb, J. R. Cheeseman, G. Scalmani, V. Barone, B. Mennucci, G. A. Petersson, H. Nakatsuji, M. Caricato, X. Li, H. P. Hratchian, A. F. Izmaylov, J. Bloino, G. Zheng, J. L. Sonnenberg, M. Hada, M. Ehara, K. Toyota, R. Fukuda, J. Hasegawa, M. Ishida, T. Nakajima, Y. Honda, O. Kitao, H. Nakai, T. Vreven, J. A. Montgomery, Jr., J. E. Peralta, F. Ogliaro, M. Bearpark, J. J. Heyd, E. Brothers, K. N. Kudin, V. N. Staroverov, R. Kobayashi, J. Normand, K. Raghavachari, A. Rendell, J. C. Burant, S. S. Iyengar, J. Tomasi, M. Cossi, N. Rega, J. M. Millam, M. Klene, J. E. Knox, J. B. Cross, V. Bakken, C. Adamo, J. Jaramillo, R. Gomperts, R. E. Stratmann, O. Yazyev, A. J. Austin, R. Cammi, C. Pomelli, J. W. Ochterski, R. L. Martin, K. Morokuma, V. G. Zakrzewski, G. A. Voth, P. Salvador, J. J. Dannenberg, S. Dapprich, A. D. Daniels, Ö. Farkas, J. B. Foresman, J. V. Ortiz, J. Cioslowski, and D. J. Fox, Gaussian, Inc., Wallingford CT, 2009.

(32) Tao, J.; Perdew, J. P.; Staroverov, V. N.; Scuseria, G. E. *Phys. Rev. Lett.* **2003**, *91* (14), 146401

(33) Weigend, F. *Phys. Chem. Chem. Phys.* **2006**, *8* (9), 1057–1065.

(34) Weigend, F.; Ahlrichs, R. *Phys. Chem. Chem. Phys.* **2005**, *7* (18), 3297–3305.

(35) Marenich, A. V.; Cramer, C. J.; Truhlar, D. G. *J. Phys. Chem. B* **2009**, *113* (18), 6378–6396.

(36) Álvarez-Moreno, M.; de Graaf, C.; Lopez, N.; Maseras, F.; Poblet, J.M.; Bo, C. *J. Chem. Inf. Model.* **2015**, *55*, 95-103.
